# Leaf transcriptomes from C_3_, C_3_-C_4_ intermediate, and C_4_*Neurachne* species give insights into C_4_ photosynthesis evolution

**DOI:** 10.1093/plphys/kiae424

**Published:** 2024-08-16

**Authors:** Maximilian Lauterbach, Andrea Bräutigam, Harmony Clayton, Montserrat Saladié, Vivien Rolland, Terry D Macfarlane, Andreas P M Weber, Martha Ludwig

**Affiliations:** School of Molecular Sciences, University of Western Australia, Perth, WA 6009, Australia; Faculty of Biology, Bielefeld University, Bielefeld 33501, Germany; School of Molecular Sciences, University of Western Australia, Perth, WA 6009, Australia; School of Molecular Sciences, University of Western Australia, Perth, WA 6009, Australia; Commonwealth Scientific and Industrial Research Organisation, Black Mountain Laboratories, Canberra, ACT 2601, Australia; School of Molecular Sciences, University of Western Australia, Perth, WA 6009, Australia; Department of Biodiversity, Conservation and Attractions, Biodiversity and Conservation Science Division, Western Australian Herbarium, Perth, WA 6152, Australia; Institute for Plant Biochemistry, Heinrich-Heine-University, Duesseldorf 40225, Germany; School of Molecular Sciences, University of Western Australia, Perth, WA 6009, Australia

## Abstract

The C_4_ photosynthetic pathway is hypothesized to have evolved from the ancestral C_3_ pathway through progressive changes in leaf anatomy and biochemistry with extant C_3_-C_4_ photosynthetic intermediate species representing phenotypes between species demonstrating full C_3_ and full C_4_ states. The Australian endemic genus *Neurachne* is the only known grass group that contains distinct, closely related species that carry out C_3_, C_3_-C_4_ intermediate, or C_4_ photosynthesis. To explore and understand the molecular mechanisms underlying C_4_ photosynthesis evolution in this genus, leaf transcriptomes were generated from two C_3_, three photosynthetic intermediate (proto-Kranz, C_2_-like, and C_2_), and two C_4_*Neurachne* species. The data were used to reconstruct phylogenetic relationships in *Neurachne*, which confirmed two independent C_4_ origins in the genus. Relative transcript abundances substantiated the photosynthetic phenotypes of individual species and highlighted transcriptional investment differences between species, including between the two C_4_ species. The data also revealed proteins potentially involved in C_4_ cycle intermediate transport and identified molecular mechanisms responsible for the evolution of C_4_-associated proteins in the genus.

## Teaser

Leaf transcriptomes of C_3_, C_3_-C_4_ intermediate, and C_4_*Neurachne* species give insights into the molecular mechanisms used during the evolution of 2 independent C_4_ origins in the genus.

## Introduction

C_4_ photosynthesis is a carbon concentrating mechanism (CCM) that, despite its complex nature, independently evolved from the ancestral C_3_ photosynthetic pathway multiple times in a wide range of mono- and eudicot plant lineages (Sage 2016). While the reactions of carbon assimilation and reduction occur in leaf mesophyll (M) cells of plants using C_3_ photosynthesis, in most species conducting C_4_ photosynthesis, the CCM is facilitated by a spatial division of photosynthetic reactions between two cell types: CO_2_ assimilation in M cells and carbon (C) reduction in a layer of cells surrounding the vascular bundle. In C_4_ M cells, carbonic anhydrase (CA) converts atmospheric CO_2_ to bicarbonate that is used by phospho*enol*pyruvate carboxylase (PEPC) to produce the 4-C organic acid, oxaloacetate (OAA), which is immediately converted to malate and/or aspartate (Hatch 1987). These 4-C compounds diffuse through plasmodesmata into the cells of the vascular sheath (VS), where they are decarboxylated, with the released CO_2_ being fixed by ribulose-1,5-bisphosphate carboxylase/oxygenase (Rubisco), which is found only in the VS, and carbohydrates made through the Calvin–Benson–Bassham (CBB) cycle. The 3-C compound released by the decarboxylation event diffuses to the M cells where it is converted to PEP by pyruvate orthophosphate dikinase (PPDK) for a subsequent round of the C_4_ acid transfer cycle (Hatch 1987). The arrangement of M cells surrounding the VS cell layer, a leaf anatomy known as Kranz anatomy (Haberlandt 1884), is also important for the efficient functioning of the CCM in two-celled C_4_ species (Hatch 1987). The little to no exposure of VS cells to leaf intercellular airspaces, oftentimes modification of VS cell walls and/or the positioning of organelles in the cells, the spatial separation of C_4_-associated enzymes, and the use of PEPC, a primary carboxylase that does not recognize O_2_ unlike Rubisco, enable the concentration of CO_2_ in VS cells to be up to 20 times that of the surrounding M cells (Jenkins et al. 1989). Consequently, the carboxylase activity of Rubisco is favored over its oxygenase activity, and loss of previously fixed C through photorespiration is greatly reduced. This is in stark contrast to the scenario in C_3_ plants where Rubisco is the primary carboxylase and, under current atmospheric conditions, photorespiration is 26% of the CO_2_ assimilation rate (Sharkey 1988). These modifications to leaf anatomy and CO_2_ assimilation give C_4_ plants growth and survival advantages over C_3_ species in hot, dry, and/or high-light environments (Osborne and Freckleton 2009).

Due to the spatial separation of the biochemical reactions of Kranz-type C_4_ photosynthesis, cell-preferential expression of the enzymes is a hallmark of the pathway. For example, the C_4_-associated CA and PEPC are expressed preferentially in the M, while the enzymes involved in decarboxylation of the 4-C organic acids and Rubisco show preferential expression in VS cells (Hatch 1987; Sage et al. 2012; Furbank 2016). In addition, movement of intermediates of CO_2_ assimilation is increased in C_4_ plants relative to their C_3_ ancestors due to the involvement of two cell types and is an absolute requirement for a robust C_4_ CCM (Weber and von Caemmerer 2010; von Caemmerer and Furbank 2016). While many of the shuttled metabolites and transport proteins involved are known, some are yet to be identified (Weber and von Caemmerer 2010; Schlüter et al. 2016; Schlüter and Weber 2020; Borghi et al. 2022).

All known genes encoding C_4_-associated proteins have been co-opted from C_3_ species with modifications to the ancestral genes that have resulted in changes to regulatory mechanisms and the encoded proteins (Hibberd and Covshoff 2010; Weber and von Caemmerer 2010; Gowik and Westhoff 2011; Langdale 2011; Sage et al. 2012; Ludwig 2013; Heyduk et al. 2019; Schlüter and Weber 2020; Ludwig et al. 2024). Gene duplication followed by neofunctionalization and/or subfunctionalization (Monson 2003; Ludwig 2013; Emms et al. 2016; Niklaus and Kelly 2019) and lateral gene transfer events (Christin et al. 2012a; Dunning et al. 2019; Hibdige et al. 2021) are mechanisms that have aided the evolution of a C_4_ syndrome in multiple lineages. For a number of C_4_-associated enzymes, amino acid residues have been identified that have undergone positive selection in multiple C_4_ lineages (Christin et al. 2007, 2009, 2012b; Besnard et al. 2009; Rosnow et al. 2014, 2015; Goolsby et al. 2018; Khoshravesh et al. 2020a; Yamamoto et al. 2022). All of the above molecular modifications support the view that C_4_ photosynthesis is a paradigm of convergent evolution.

While a C_4_ syndrome independently evolved in more than 60 angiosperm lineages (Sage 2016), some of these groups are of particular interest as they comprise more than one independent C_4_ origin and/or species that show photosynthetic phenotypes between that of a full C_3_ and a full C_4_ species (Sage et al. 2012). Such C_3_-C_4_ photosynthetic intermediate species may be more C_3_-like (proto-Kranz) or have nearly full C_4_ leaf anatomy and/or biochemistry (C_4_-like). Other C_3_-C_4_ photosynthetic intermediates demonstrate a photorespiratory CO_2_ pump (C_2_ photosynthesis) in which most or all photorespiratory glycine is decarboxylated in the mitochondria of VS cells due to the restricted localization of glycine decarboxylase (GDC) to that cell type. A recent study using *Flaveria*, a eudicot genus rich in C_3_-C_4_ intermediate species as well as containing full C_3_ and full C_4_ species, described six functional clusters of photosynthetic phenotypes: full C_3_, sub-C_2_, full C_2_, enriched C_2_, sub-C_4_, and full C_4_ (Adachi et al. 2023). While a C_3_-C_4_ intermediate phenotype does not appear to represent an evolutionary intermediate state in some groups (Christin et al. 2011; Walsh et al. 2023), in other taxa, phylogenetic reconstructions support such a position and C_3_-C_4_ intermediate species have facilitated the investigation of potential evolutionary steps from the ancestral C_3_ state to a complete C_4_ syndrome (Monson et al. 1984; Rawsthorne 1992; Sage et al. 2012; Heckmann et al. 2013; Williams et al. 2013).

The genus *Neurachne* (Poaceae, subfamily Panicoideae, tribe Paniceae, subtribe Neurachninae; Soreng et al. 2022) is an important and unique taxon for studying the evolution of C_4_ photosynthesis as it exhibits an unusually high diversity of photosynthetic biochemistries on the spectrum of C_3_ to C_4_ photosynthesis within a small group of distinct, closely related species (Hattersley et al. 1982, 1986; Hattersley and Roksandic 1983; Hattersley and Stone 1986; Moore and Edwards 1989; Christin et al. 2012b; Khoshravesh et al. 2020b). The genus consists of three C_3_ species, *N. alopecuroidea*, *N. queenslandica*, and *N. tenuifolia*; one proto-Kranz species, *N. annularis*; one C_2_-like species with an incipient photorespiratory CO_2_ pump, *N. lanigera*; one full C_2_ species, *N. minor*; and two C_4_ species, *N. muelleri* and *N. munroi*, that show independent evolutionary origins (Christin et al. 2012b; Khoshravesh et al. 2020b) and use NADP-malic enzyme (ME) as the major decarboxylating enzyme (Hattersley and Stone 1986). Except for *N. alopecuroidea*, which is found in temperate regions of Western Australia, South Australia, and Victoria, all *Neurachne* species inhabit arid and subarid zones of Australia (Prendergast and Hattersley 1985; Christin et al. 2012b). *Neurachne* species grow as tufted grasses, with horizontal rhizomes, and may form tussocks under favorable conditions (Blake 1972; Macfarlane 2007). The C_4_*N. muelleri* is a distinctive member of the genus as it propagates vegetatively via stolons (Blake 1972; Prendergast and Hattersley 1985).

*Neurachne* species exhibit Neurachneoid leaf anatomy (Dengler and Nelson 1999; Edwards and Voznesenskaya 2011) in which the vascular bundles are surrounded by two VS layers; an outer parenchymatous sheath (PS) and an inner thick-walled mestome sheath (MS; Hattersley et al. 1982; 1986; Khoshravesh et al. 2020b). The allocation of organelles to *Neurachne* leaf PS cells is relatively low compared to that of the M and MS regardless of photosynthetic type (Khoshravesh et al. 2020b). By contrast, chloroplast investment in MS cells of C_2_*N. minor* and the two C_4_ species is greater than that of the C_2_-like, proto-Kranz, and C_3_ species, while MS mitochondrial allocation is greatest in the C_2_ species as expected due to its photorespiratory CO_2_ pump activity (Khoshravesh et al. 2020b). The preferential localization of Rubisco (Khoshravesh et al. 2020b, Supplemental data) to C_4_*Neurachne* MS chloroplasts and the P subunit protein of GDC to C_4_ and C_2_ MS mitochondria (Khoshravesh et al. 2020b) indicate the MS layer is the functional equivalent of the BS in other C_2_ and C_4_ species.

Here, we report leaf transcriptome data for seven *Neurachne* species that represent stages along the C_3_ to C_4_ evolutionary continuum: two C_3_ species, *N. alopecuroidea* and *N. tenuifolia*; the proto-Kranz and C_2_-like species, *N. annularis* and *N. lanigera*, respectively; C_2_*N. minor*; and the two C_4_ species, *N. muelleri* and *N. munroi*. Using these data, we inferred their phylogenetic relationships within *Neurachne* and investigated transcriptional investment and differences in gene expression patterns among the seven species. The expression data supported the distinct photosynthetic phenotypes demonstrated in the genus, including the two independent origins of C_4_ photosynthesis. Differences in molecular mechanisms that enabled the evolution of C_4_ syndromes in *N. muelleri* and *N. munroi* were revealed as were amino acid residues that were positively selected during the evolution of C_4_*Neurachne* PEPC and NADP-ME isoforms.

## Results

### Quality filtering of *Neurachne* leaf RNA-Seq reads

RNA-Seq generated between 3.95 and 8.74 million 100 base pair (bp) paired-end sequencing reads per *Neurachne* leaf sample, with at least 3.88 million reads per sample remaining after quality filtering (Supplementary Table S1). The number of assembled contigs for the seven *Neurachne* species ranged from 85,808 in *N. tenuifolia* to 149,830 in *N. alopecuroidea* (Supplementary Table S1) and was reduced via CD-HIT-EST to between 73,984 and 121,957 contig clusters. After pairwise orthology assessment against the *Setaria italica* genome, a dataset of 11,580 genes that were expressed in all replicates of all seven *Neurachne* species remained (Supplementary Table S2). Downstream analyses were limited to this dataset. Analysis of transcriptome completeness using BUSCO v.3.0 (Benchmarking Universal Single-Copy Orthologs) with the Liliopsida odb10 dataset (Simão et al. 2015; Kriventseva et al. 2019) indicated 78% to 87% complete and 4.1% to 11.1% fragmented matches across all *Neurachne* leaf transcriptomes (Supplementary Table S3). The values for missing BUSCO matches were also similar for all transcriptomes (Supplementary Table S3) and likely reflect genes expressed at different developmental stages or organs other than mature leaves (Manni et al. 2021). No consistent differences in the percentages of BUSCO duplicated matches were seen between diploid (*N. tenuifolia*, *N. annularis*, and *N. lanigera*; Christin et al. 2012b) and polyploid (*N. alopecuroidea*, *N. minor*, *N. munroi*, and *N. muelleri*; Christin et al. 2012b) species (Supplementary Table S3) potentially due to multiple transcripts mapping to the same gene.

### Reconstruction of relationships within *Neurachne* using a phylotranscriptomics approach

**Figure 1.**
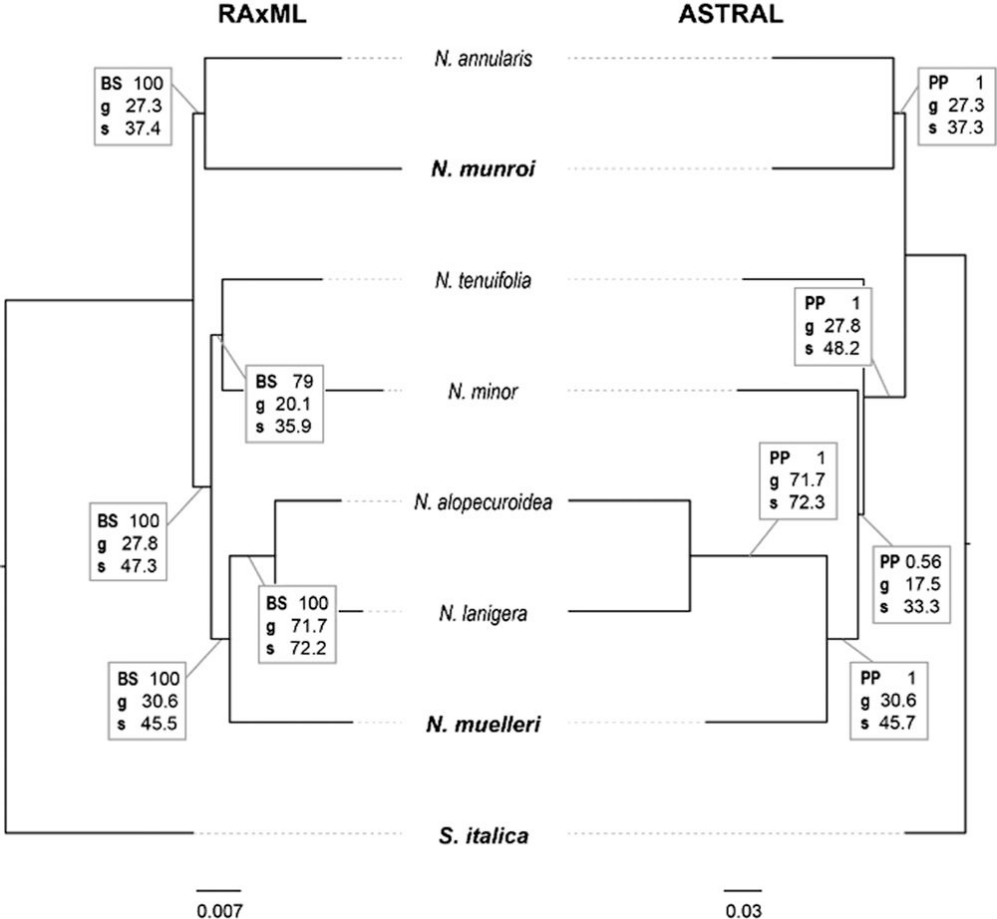
Inference of phylogenetic relationships within *Neurachne*. Trees were inferred using a supermatrix of all markers under maximum likelihood in RAxML (left), and Bayesian inference in ASTRAL (right). *Setaria italica* was used as the outgroup taxon in all analyses. Numbers in boxes indicate: BS support values (left) or PP (right); gene concordance factor (g); site concordance factor (s). Bold text indicates species conducting C_4_ photosynthesis. Scale bars below trees indicate number of substitutions per site.

Both concatenated and species tree methods, inferred with RAxML and ASTRAL, respectively, were conducted to reconstruct species relationships in *Neurachne* using the RNA-Seq results (Fig. 1). The dataset used for the supermatrix approach for final tree inference included only regions of ≥1,200 bp length, which resulted in 671 orthologous sequences present in all species with 1,121,163 aligned positions in total, and an overall matrix occupancy of 80%. Both analyses revealed two clades within *Neurachne* that shared a common ancestor where one clade included *N. annularis* (proto-Kranz) and *N. munroi* (C_4_) and had maximum likelihood bootstrap (BS) and posterior probability (PP) values of 100 and 1, respectively (Fig. 1). The second clade comprised all other *Neurachne* species (BS = 100; PP = 1). Within this second clade, incongruences were found between the two tree inference methods, neither of which was well supported. In the maximum likelihood tree, *N. minor* (C_2_) and *N. tenuifolia* (C_3_) formed a weakly supported sister group (BS = 79) and together formed a well-supported sister group (BS = 100) to a clade that included *N. alopecuroidea* (C_3_), *N. lanigera* (C_2_-like), and *N. muelleri* (C_4_) (BS = 100). By contrast, in the supermatrix tree based on Bayesian inference, *N. tenuifolia* was sister to a clade comprising *N. minor*, *N. alopecuroidea*, *N. lanigera*, and *N. muelleri* (PP = 0.56).

To better understand what caused the incongruences in the tree topologies, for each branch of the topologies resulting from RAxML and ASTRAL analyses, gene concordance (gCF) and site concordance (sCF) factors were calculated. Gene concordance is the percentage of gene trees containing a particular branch, while sCF is the percentage of alignment sites supporting a particular branch in the reference tree. The highest gCF and sCF scores were found on the branch leading to *N. alopecuroidea* and *N. lanigera* (gCF = 71.7, sCF = 72.2/72.3; Fig. 1; Supplementary Table S4). The branches with low BS or PP support also had low gCF and sCF values. In the RAxML tree, this was the branch leading to *N. minor* and *N. tenuifolia* (BS = 79, gCF = 20.1, sCF = 35.9; Fig. 1; Supplementary Table S4), while in the ASTRAL tree, the branch leading to *N. minor*, *N. alopecuroidea* and *N. lanigera*, and *N. muelleri* had the lowest values (PP = 0.56, gCF = 17.5, sCF = 33.3; Fig. 1; Supplementary Table S4). The branches leading to these taxa are relatively short and likely reflect a lack of informative sites for phylogenetic inference or suggest relationships among species are reticulate rather than dichotomous. Consequently, it remains uncertain whether *N. minor* and *N. tenuifolia* are in a sister group relationship (Fig. 1, RAxML tree) or whether *N. minor* is sister to the group comprising *N. alopecuroidea*, *N. lanigera*, and *N. muelleri*, with *N. tenuifolia* sister to this lineage (Fig. 1, ASTRAL tree). The second lowest gCF and sCF scores were found for the branch in both trees leading to *N. annularis* and *N. munroi* (gCF = 27.3, sCF = 37.3/37.4; Fig. 1; Supplementary Table S4). Again, these branches are comparatively short, which may be responsible for the low gCF and sCF values. The gene discordance factor (gDF) and site discordance factor (sDF) were relatively high for these branches, indicating that the support for alternative topologies was high (Supplementary Table S4). The second and third most common topology showed *N. annularis* (gDF_1 = 18.1, sDF_1 = 30.1) or *N. munroi* (gDF_2 = 17.4, gSF_2 = 32.5/32.6) as sister to all other species within *Neurachne*, respectively (Supplementary Table S4). Results of the phylogenetic network inference done with SplitsTree4 showed a star-like structure with species clearly distinct from each other but relationships among species remaining vague (Supplementary Fig. S1).

### Differences in transcript profiles between *Neurachne* species

**Figure 2.**
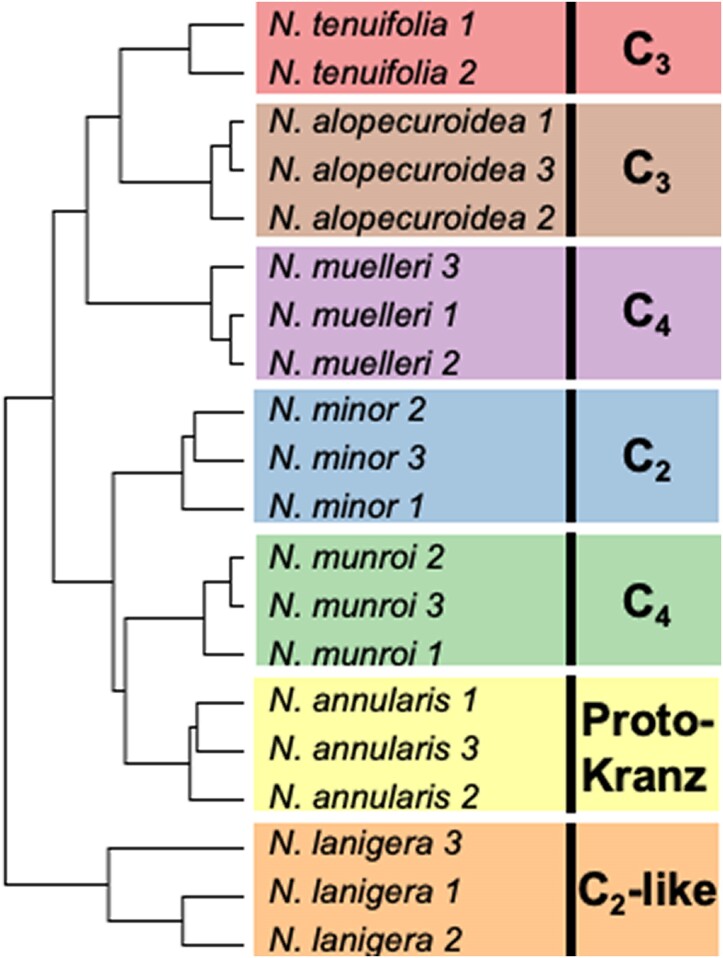
Hierarchical clustering of all transcribed genes in *Neurachne* leaf samples using Pearson Correlation after log_2_ transformation of read counts. For each species, leaves from three individuals were used except for *N. tenuifolia* where only two individuals were available for RNA-Seq analysis (see Materials and methods). The type of photosynthetic pathway used by the species is indicated at the right of the figure.

The results of hierarchical cluster analysis using *Neurachne* transcript abundances showed the replicates of each species grouped together (Fig. 2). The trend in hierarchical clustering only partly reflected previously published phylogenetic relationships (Christin et al. 2012b) and that reconstructed in this study as described above (Fig. 1). For example, phylogenetic analyses indicated the C_2_-like species *N. lanigera* is closely related to *N. alopecuroidea* and *N. muelleri* (Fig. 1; Christin et al. 2012b), while the clustering of *N. lanigera* transcript data appeared to be different to that of all other *Neurachne* species investigated in the current study (Fig. 2). The two C_4_ species do not group together based on leaf total transcript data. Instead, C_4_*N. muelleri* grouped with C_3_*N. alopecuroidea* and *N. tenuifolia*, while C_4_*N. munroi* clustered with the proto-Kranz *N. annularis* and the C_2_ species *N. minor* (Fig. 2). This topology is consistent with phylogenetic reconstructions found in this and a previous study (Fig. 1; Christin et al. 2012b).

Principal component analysis (PCA; Supplementary Fig. S2) overall supported the hierarchical clustering results shown in Fig. 2; replicates of the same species tended to group together, with the three individuals of *N. lanigera* showing a higher variation than that of the other species (Supplementary Fig. S2). The first two components explained 37.94% of the total variance, while 51.55% of the total variance was explained by the first three components (Supplementary Fig. S2). As observed in the hierarchical clustering analysis (Fig. 2), combinations of the first principal component with either of the other two components (Supplementary Fig. S2, A and C) did not group the species as in reconstructed phylogenies (Fig. 1; Christin et al. 2012b) nor according to photosynthetic type. However, a combination of principal components 2 and 3 grouped the two C_4_ species (*N. munroi* and *N. muelleri*) together and the C_3_ species (*N. alopecuroidea* and *N. tenuifolia*) together (Supplementary Fig. S2B). In addition, the species with intermediate photosynthetic biochemistries (proto-Kranz *N. annularis*, C_2_-like *N. lanigera*, C_2_*N. minor*) grouped together between the full C_3_ and full C_4_ species with this combination of principal components (Supplementary Fig. S2B).

**Figure 3.**
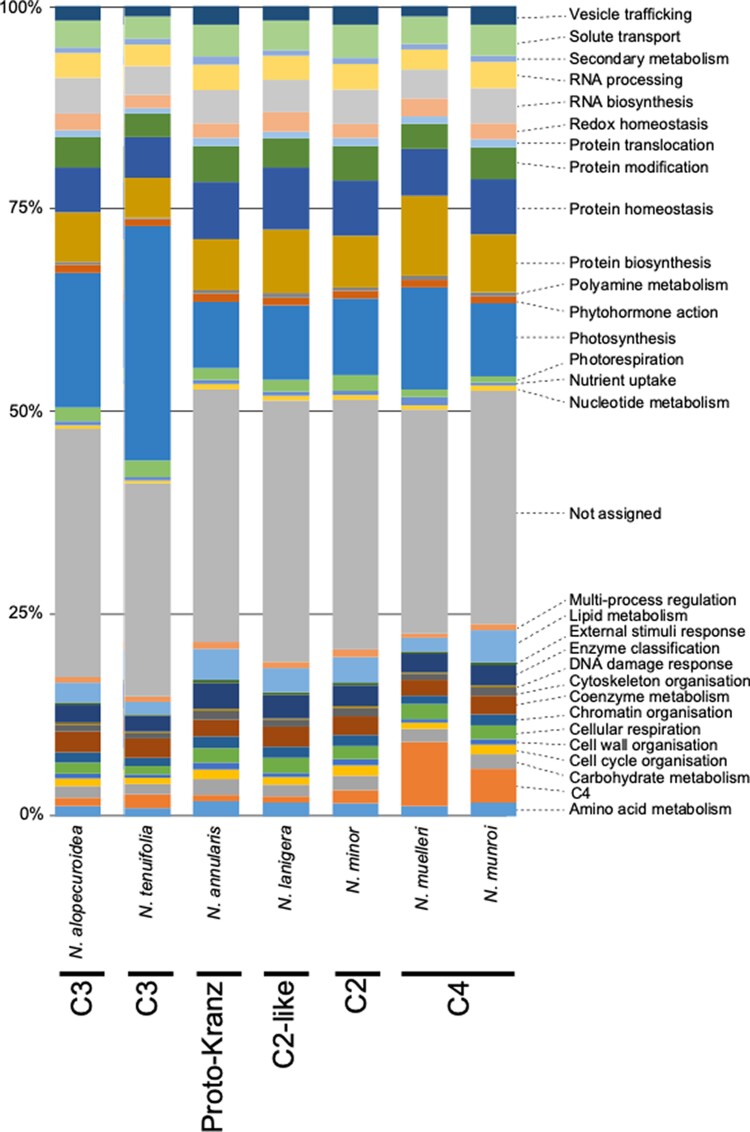
Distribution of transcriptional investment of *Neurachne* leaf transcripts. Transcriptional investment is defined as the percentage of transcripts belonging to a particular MapMan4 category and the two additional categories “C_4_” and “photorespiration”.

When looking at the transcriptional investment into different biochemical functions, based on MapMan4 categories with the addition of “C_4_” and “photorespiration”, investment in the categories “not assigned” (26.3% to 32.3%), “protein biosynthesis” (5.0% to 9.9%), and “protein homeostasis” (5.1% to 7.6%) was high in all seven *Neurachne* species (Fig. 3). Investment in the category “photosynthesis” was high in all species, but clearly differed between them: 16.6% and 28.9% in the C_3_ species *N. alopecuroidea* and *N. tenuifolia*, respectively, and between 8.2% and 12.6% in the other five species (Fig. 3). These differences were not driven by the abundance of a single transcript or those from a few genes, but instead by differential expression of many genes belonging to the category “photosynthesis” (Supplementary Table S2). “C_4_” was the fourth highest category in *N. muelleri* (7.9%) and sixth highest in *N. munroi* (4.2%), while as expected, transcriptional investment was relatively low in this category in the non-C_4_ species (0.8% to 1.7%). Transcriptional investment in “photorespiration”, by contrast, was higher in the C_3_, proto-Kranz, and C_2_-like species (1.5% to 2.1%), as well as C_2_*N. minor* (2.0%), compared to the two C_4_ species (0.8% to 0.9%). Investment in the category “nutrient uptake” was slightly higher in C_4_*N. muelleri* (1.1%) compared to all the other species (0.3% to 0.5%). This difference was driven by transcripts encoding a homolog of an Arabidopsis (*Arabidopsis thaliana*) copper (Cu) chaperone and the transcripts from several genes encoding proteins involved in sulfur (S) assimilation (Supplementary Table S2; Supplementary Fig. S3).

### Transcripts encoding proteins associated with C_4_ photosynthesis and photorespiration and candidate C_4_-associated proteins in *Neurachne*

**Figure 4.**
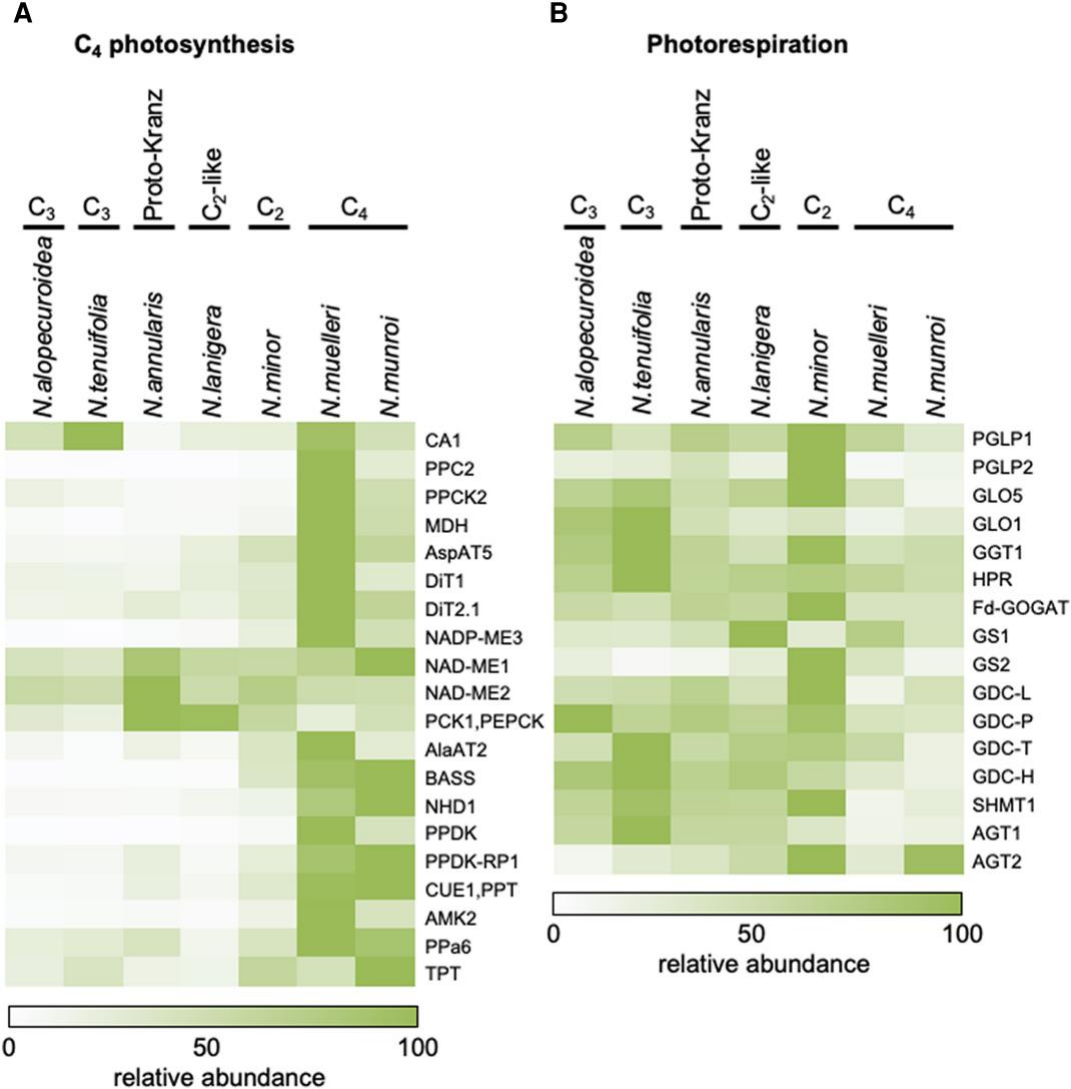
Relative abundance of transcripts encoding proteins associated with C_4_ photosynthesis and photorespiration in *Neurachne* leaf transcriptomes. Heat map shows normalized, relative transcript abundance of genes encoding major proteins of **A)** C_4_ photosynthesis and **B)** photorespiration. AGT, serine–glyoxylate aminotransferase; Ala-AT, alanine aminotransferase; AMK, adenosine monophosphate kinase; Asp-AT, aspartate aminotransferase; BASS, bile acid sodium symporter family protein 2; CA, *β*-carbonic anhydrase; CUE/PPT phospho*enol*pyruvate phosphate translocator; DiT, dicarboxylate transporter; Fd-GOGAT, ferredoxin-dependent glutamate synthase; GDC, glycine decarboxylase; GGT, glutamate–glyoxylate aminotransferase; GLO, glycolate oxidase; GS, glutamine synthetase; HPR, hydroxypyruvate reductase; MDH, malate dehydrogenase; NAD-ME, NAD-malic enzyme; NADP-ME, NADP-malic enzyme; NDH, sodium:hydrogen antiporter; PCK/PEPC-K phospho*enol*pyruvate carboxykinase; PGLP, phosphoglycolate phosphatase; PPa, pyrophosphorylase; PPC, phospho*enol*pyruvate carboxylase; PPCK, phospho*enol*pyruvate carboxylase kinase; PPDK, pyruvate orthophosphate dikinase; PPDK-RP, pyruvate orthophosphate dikinase regulatory protein; SHMT, serine hydroxymethyltransferase; TPT, triose-phosphate phosphate translocator.

Transcripts encoding the C_4_-associated adenosine monophosphate kinase (AMK), aspartate aminotransferase (Asp-AT), bile acid–sodium symporter family protein 2 (BASS2), dicarboxylate transporters (DiTs), malate dehydrogenase (MDH), NADP-ME, sodium–hydrogen antiporter (NHD), PEPC, PPDK, and PEP phosphate translocator (PPT) were significantly (FDR *q* ≤ 0.01) more abundant in the two C_4_ species compared to the C_3_ species [log_2_ fold change (FC) between 1.3 and 9.5; Fig. 4A; Supplementary Table S2]. Moreover, these transcripts were more abundant in leaves from *N. muelleri* than *N. munroi*. Interestingly, analysis of differentially expressed genes among *Neurachne* species showed the numbers are highest when comparing the two C_4_ species: 1,789 and 2,109 transcripts were significantly higher and lower, respectively, in *N. munroi* compared to *N. muelleri* (Supplementary Table S5). Levels of transcripts encoding most of the C_4_-associated proteins were higher in C_2_*N. minor* than in the C_3_, proto-Kranz, and C_2_-like species (Fig. 4A; Supplementary Table S2), with transcripts encoding alanine AT (Ala-AT), AMK, BASS2, NADP-ME, PEPC, and PPDK significantly more abundant (log_2_FC between 1.2 and 4.5; Fig. 4A; Supplementary Table S2).

Transcripts coding for photorespiratory proteins were relatively low in the two C_4_*Neurachne* species; however, only mRNAs encoding serine–glyoxylate AT (AGT1) and serine hydroxymethyltransferase (SHMT) were significantly lower in *N. munroi* and *N. muelleri* compared to the non-C_4_ species (log_2_FC between 1.4 and 3.1; Fig. 4B; Supplementary Table S2). By contrast, transcripts encoding many of the proteins involved in photorespiration were highly abundant in C_2_*N. minor* and C_3_*N. tenuifolia* (Fig. 4B; Supplementary Table S2).

**Figure 5.**
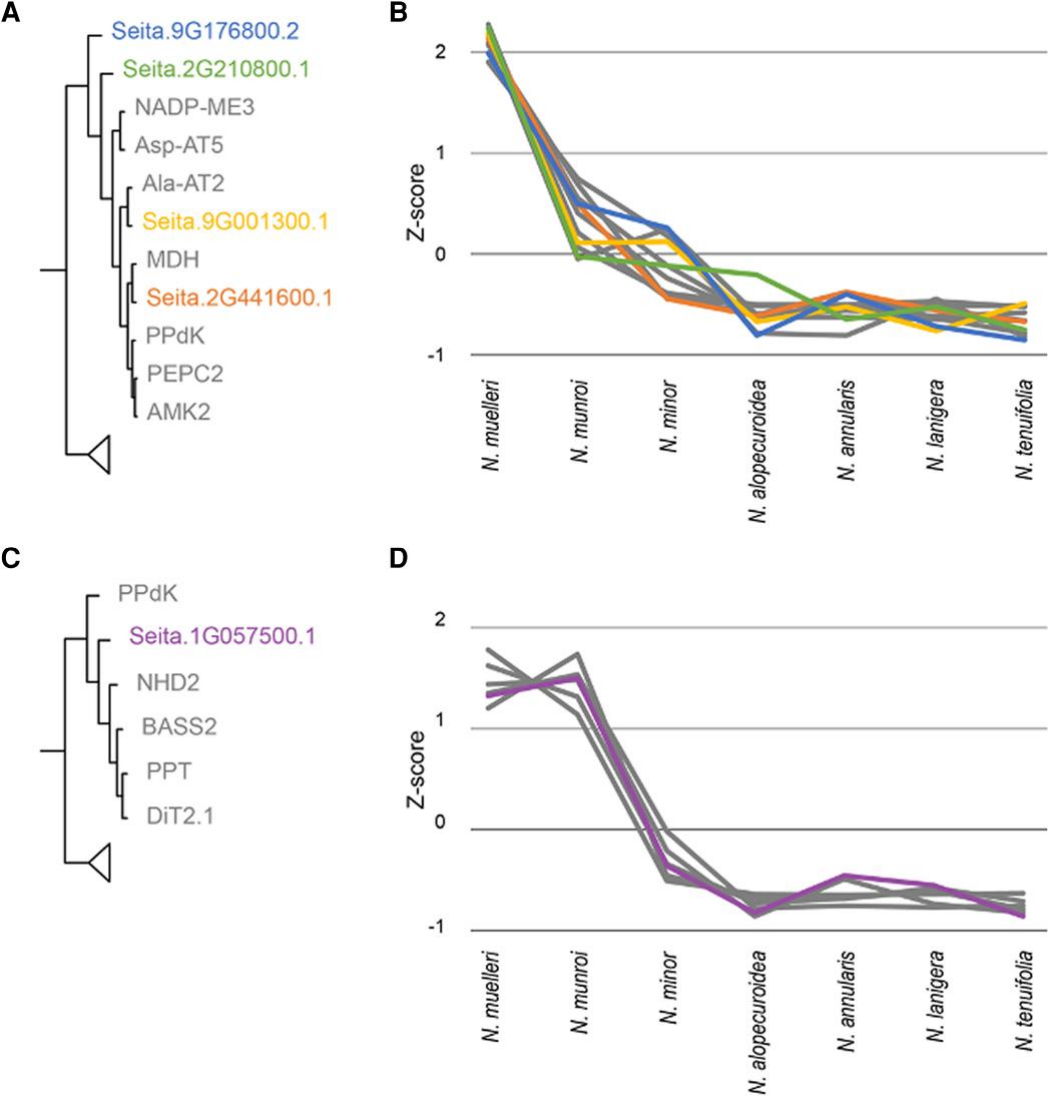
Genes demonstrating co-expression with those encoding C_4_-associated proteins. Five genes (green, orange, yellow, blue, and purple) show similar expression profiles to those of genes known to encode proteins involved in C_4_ photosynthesis (grey). **A)** Hierarchical clustering of 469 genes represented in Cluster C12 (Supplementary Fig. S4; a subset of 11 genes is shown here). **B)** Transcript abundance (shown as *z*-scores) of the 11 genes shown in **A)** in seven *Neurachne* species (line colors correlate with gene designations in **A)**). **C)** Hierarchical clustering of 469 genes represented in Cluster C12 (Supplementary Fig. S4; a subset of six genes is shown here). **D)** Transcript abundance (shown as *z*-scores) of the six genes shown in **C)** for seven *Neurachne* species (line colors correlate with gene designations in **C)**).

From the dataset of 11,580 genes that were expressed in all replicates of all seven *Neurachne* species, Clust grouped 6,050 genes into 18 different clusters (Supplementary Fig. S4). One of these clusters, cluster C12, was of particular interest because it comprised 469 genes, many of which encode proteins known to be involved in C_4_ photosynthesis (e.g. Ala-AT, BASS, NADP-ME, PEPC), and as expected, transcripts of genes within this cluster were more abundant in the two C_4_*Neurachne* species than in the non-C_4_ species (Supplementary Fig. S4). Hierarchical clustering of the genes in Cluster C12 allowed examination of putative links between the genes encoding C_4_-associated proteins and other members of this cluster. For example, based on their expression profile, five genes clustered closely to genes coding for core C_4_-associated proteins (Fig. 5). Three of the five proteins encoded by these genes have not been fully characterized. Two show homology to products of gene families encoding cytochrome P450 (Seita.9G176800, AT1G13080) and D-alanine-D-alanine ligase (Seita.2G441600, AT3G08840; Fig. 5, A and B; Supplementary Table S2), while another (Seita.2G210800) is of unassigned function (Fig. 5, A and B; Supplementary Table S2). The expression profile of a gene encoding a homolog of mesophyll envelope protein 2 (MEP2; Seita.9G001300, AT5G23890) clusters closely with that of the C_4_-associated Ala-AT (Fig. 5, A and B). The fifth gene that shows an expression profile clustering closely with genes encoding PPDK and several C_4_-associated metabolite transporters codes for a homolog of translocon at the inner chloroplast envelope 21/permease in chloroplasts 1/chloroplast import apparatus 5 (TIC21/PIC1/CIA5; Seita.1G057500, AT2G15290; Fig. 5, C and D).

### C_4_-associated CA expression and localization

**Figure 6.**
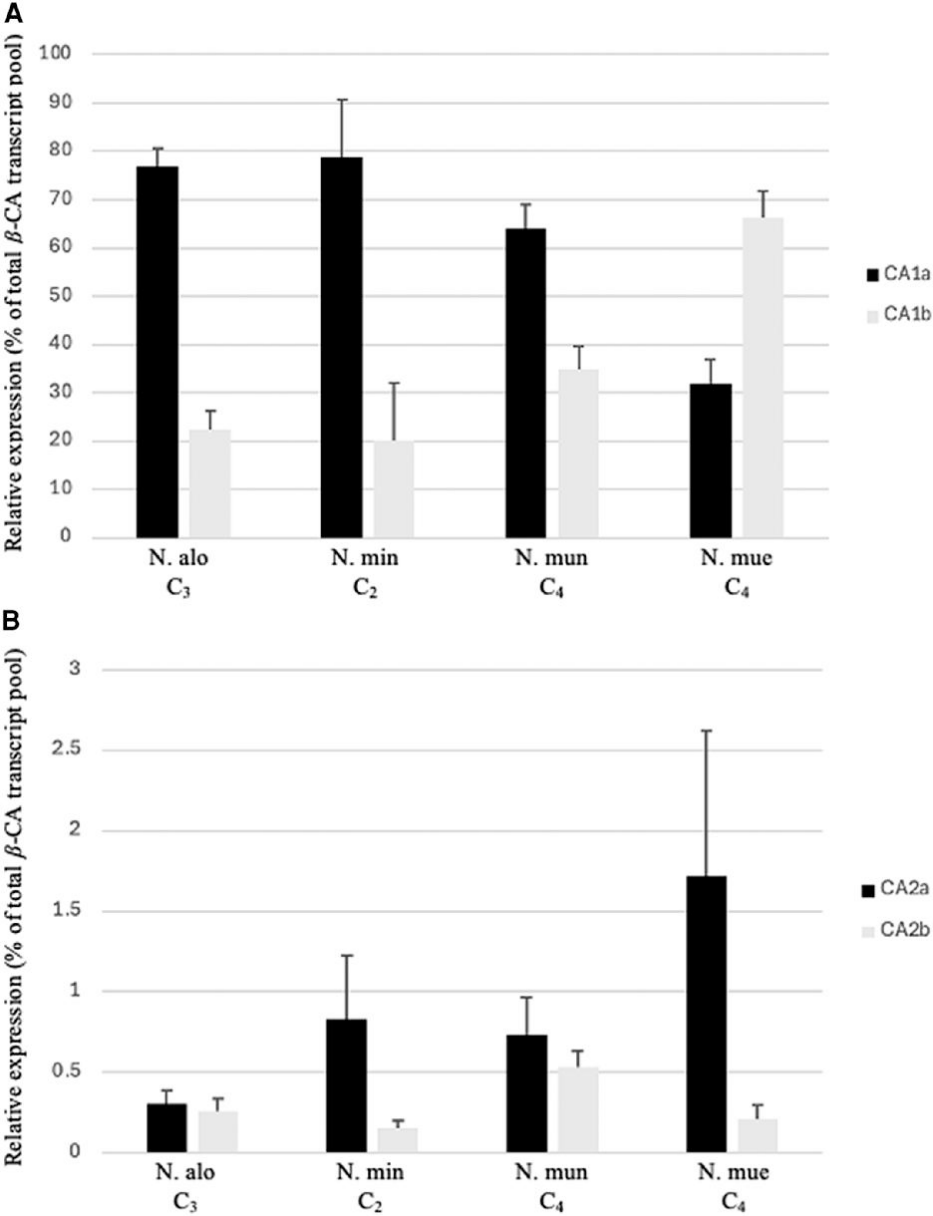
Relative abundance of transcripts encoding *β*-CA isoforms in leaves of *Neurachne* species. Transcripts encoding *β*-CA were normalized to *UBQ5* transcript abundance and are shown as percentages of the total CA transcript pool. **A)** Transcripts encoding CA1a are the most abundant transcripts in the leaves of all *Neurachne* species examined, except *N. muelleri* (N. mue) for which CA1b mRNA is the most abundant (*P* ≤ 0.01). **B)** Transcripts encoding CA2a and 2b are of low abundance in *Neurachne* leaf tissue. Data reflect measurements from three individual plants of each species, ± SD. N. alo, *N. alopecuroidea*; N. min, *N. minor;* N. mun, *N. munroi;* N. mue, *N. muelleri*.

In *Neurachne*, *β*-CA is encoded by two genes, *β*-*ca1* and *β*-*ca2*, with both producing primary transcripts that are alternatively spliced to encode the isoforms *β*-CA1a, *β*-CA1b, *β*-CA2a, and *β*-CA2b (Clayton et al. 2017). The RNA-Seq data showed transcripts from the gene encoding *β*-CA1 are the most abundant CA mRNA in all *Neurachne* species (Fig. 4A; AT5G14740; Supplementary Table S2). These results support those obtained using reverse transcription quantitative PCR (RT-qPCR) with *N. alopecuroidea*, *N. minor*, *N. munroi*, and *N. muelleri* leaf tissue (Fig. 6A; Clayton et al. 2017). In leaves of C_4_*N. muelleri*, the transcripts encoding *β*-CA1b are on average twice as abundant as transcripts coding for *β*-CA1a (*P* ≤ 0.01; Fig. 6A), which contrasts with the *β*-CA1 expression profile in leaves of C_4_*N. munroi*, as well as those of C_3_*N. alopecuroidea* and C_2_*N. minor*, where *β*-CA1a mRNA levels are approximately 2 to 4 times higher than those of *β*-CA1b mRNA (*P* ≤ 0.01; Fig. 6A; Clayton et al. 2017). For all *Neurachne* species examined, RT-qPCR assays indicated the transcripts encoding *β*-CA2a and *β*-CA2b are of very low abundance in leaves (Fig. 6B; Clayton et al. 2017).

**Figure 7.**
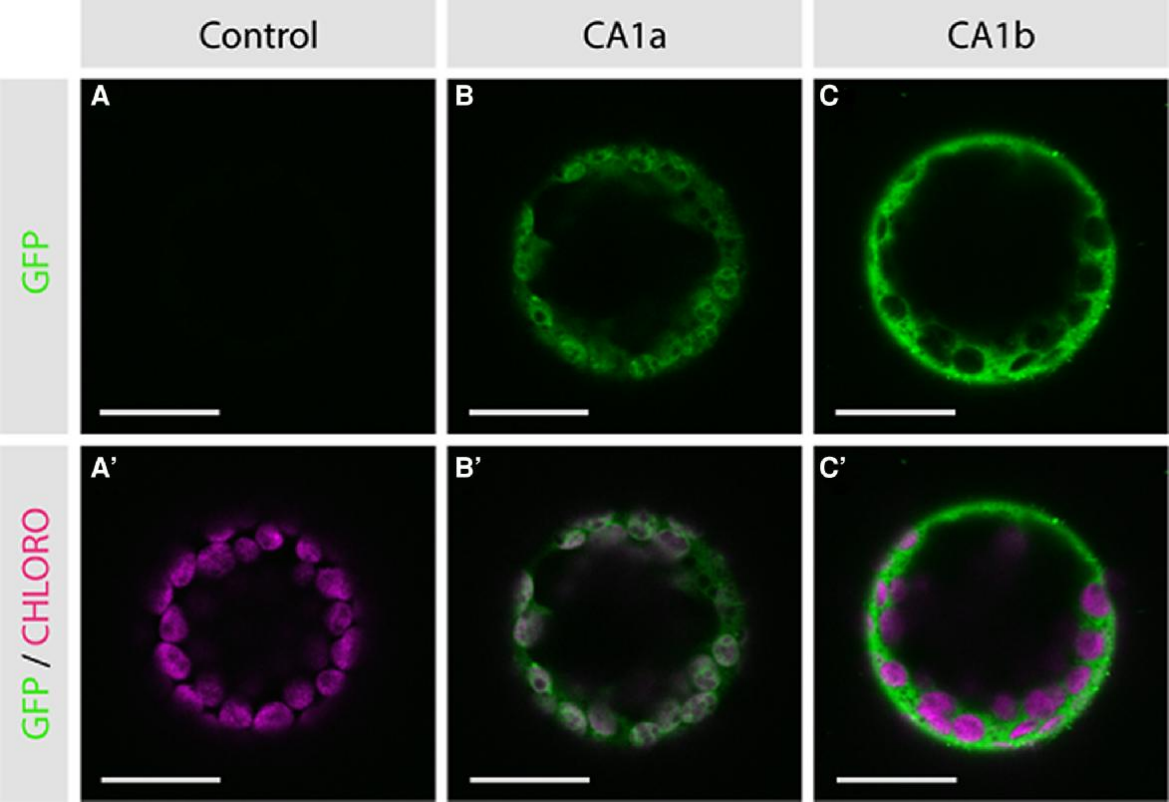
Localization of *Neurachne muelleri β*-CA 1a and 1b in tobacco (*Nicotiana benthamiana*) protoplasts. Constructs containing ORFs encoding *β*-CA 1a and 1b isoforms tagged with GFP were used to transform tobacco (*N. benthamiana*) leaves and protoplasts were isolated and imaged. The GFP signal is shown in green (A to C) and chlorophyll autofluorescence in pink (A′ to C′). No GFP signal is detected in untransformed protoplasts (A), whereas chlorophyll autofluorescence is evident in the chloroplasts (A′). The GFP signal overlaid with chlorophyll autofluorescence shows *N. muelleri* CA1a preferentially localizes to the chloroplasts (B′) while CA1b is a cytosolic isoform (C′). Bars = 20 *μ*m.

In C_4_ plants with Kranz anatomy, *β*-CA catalyzes the first reaction of the C_4_ pathway in the cytosol of M cells. Protoplasts from tobacco (*Nicotiana benthamiana*) leaves transformed with constructs encoding green fluorescence protein (GFP)–tagged *N. muelleri β*-CA1a showed this *β*-CA isoform localizes to the chloroplast, with the GFP fluorescence (Fig. 7B) overlapping the chlorophyll autofluorescence pattern (Fig. 7B′). By contrast, *N. benthamiana* protoplasts containing GFP-tagged *N. muelleri β*-CA1b showed a cytosolic location of this isoform (Fig. 7C), with the chlorophyll autofluorescence in the chloroplasts distinct from the cytosolic GFP fluorescence (Fig. 7C′). These results, taken together with those of the RT-qPCR assays, indicate *β*-CA1b is the C_4_-associated *β*-CA isoform in *N. muelleri*.

### Positively selected sites in two key C_4_ enzymes, PEPC and NADP-ME

**Figure 8.**
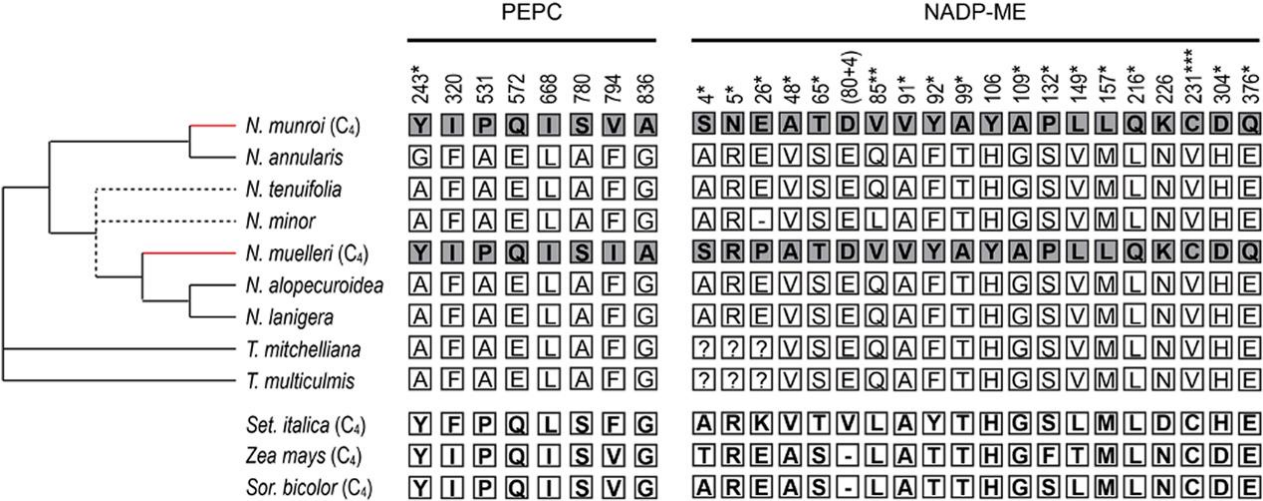
Inference of positive selection at amino acid positions in C_4_-associated PEPC and NADP-ME. Analyses were based on the cladogram shown on the left. Three different topologies were tested in positive selection analyses (see Supplementary Fig. S5) and dashed lines in the figure show parts of the tree that differed among them. Branches shown in red lead to C_4_ species that were tested against the other species. Amino acid positions with a PP ≥ 0.9 of being under positive selection are shown on the right. Positions with PP ≥ 0.95, PP ≥ 0.99, and PP = 1 are indicated with *, **, and ***, respectively. Numbering is based on maize (*Zea mays*) PEPC (ID AJ536629.1) and NADP-ME (ID AY271262.1). Amino acid sequences of the proteins from C_4_ species *S. italica* (PEPC, ID RCV21895.1; NADP-ME, ID XP_004968467.1), *Z. mays* (PEPC, ID AJ536629.1; NADP-ME, ID AY271262.1), and sorghum (*Sorghum bicolor* PEPC, ID P15804.2; NADP-ME ID XM_002454985.2) were not included in positive selection analyses, but residues are shown for comparison of the sites inferred to be under positive selection in C_4_*Neurachne*. 80 + 4 refers to the position four amino acid residues C-terminal to position 80 of the maize NADP-ME sequence at which gaps were inserted into the maize and sorghum sequences to maximize the alignment; consequently, there is no corresponding residue in the maize or sorghum sequences. Dashes (−) were included to optimize the alignment; question marks (?) indicate unresolved transcriptome sequence.

Positive selection analyses were done for 12 genes encoding proteins known to be involved in C_4_ photosynthesis. Sequence alignments of the coding regions of the 12 genes have been deposited at TreeBASE (http://purl.org/phylo/treebase/phylows/study/TB2:S25266; see also Supplementary Table S6). Analyses identified positively selected sites in the enzymes PEPC and NADP-ME along the two branches leading to the C_4_ species *N. muelleri* and *N. munroi* (Fig. 8). Considering the uncertainty of the phylogenetic positions of *N. minor* and *N. tenuifolia* described above, three different topologies were tested (Supplementary Fig. S5), and positions were inferred as positively selected, with likelihood ratio tests at a significance level of ≤0.05 (Supplementary Table S7).

Of the 969 aligned sites for PEPC, eight sites were positively selected with PP ≥ 0.9 (7 sites with 0.9 ≤ PP < 0.95, 1 site with 0.95 ≤ PP < 0.99; Fig. 8; Supplementary Table S8). At positions 243, 531, 572, and 780, the same amino acid is found in the PEPC isoforms from C_4_*Neurachne* species and the other NADP-ME–type C_4_ grasses included in the current analysis; however, none of non-C_4_ Neurachninae, including C_2_*N. minor*, shares these residues (Fig. 8). At positions 320 and 668, Ile residues are found in *N. munroi*, *N. muelleri*, maize, and sorghum (*Sorghum bicolor*) PEPC homologs, whereas the *S. italica* and non-C_4_ Neurachninae homologs contain alternative nonpolar residues, Phe and Leu, respectively (Fig. 8). A difference in nonpolar amino acids is also seen at position 836 with the two C_4_*Neurachne* PEPC homologs containing an Ala residue and all other species a Gly residue (Fig. 8). The amino acid residue at position 794 differs between the two *Neurachne* C_4_ species with Val in *N. munroi* PEPC, as in maize and sorghum, and an Ile residue in the *N. muelleri* homolog (Fig. 8). All non-C_4_ Neurachninae and the C_4_*S. italica* PEPC homologs contain Phe at position 794.

**Figure 9.**
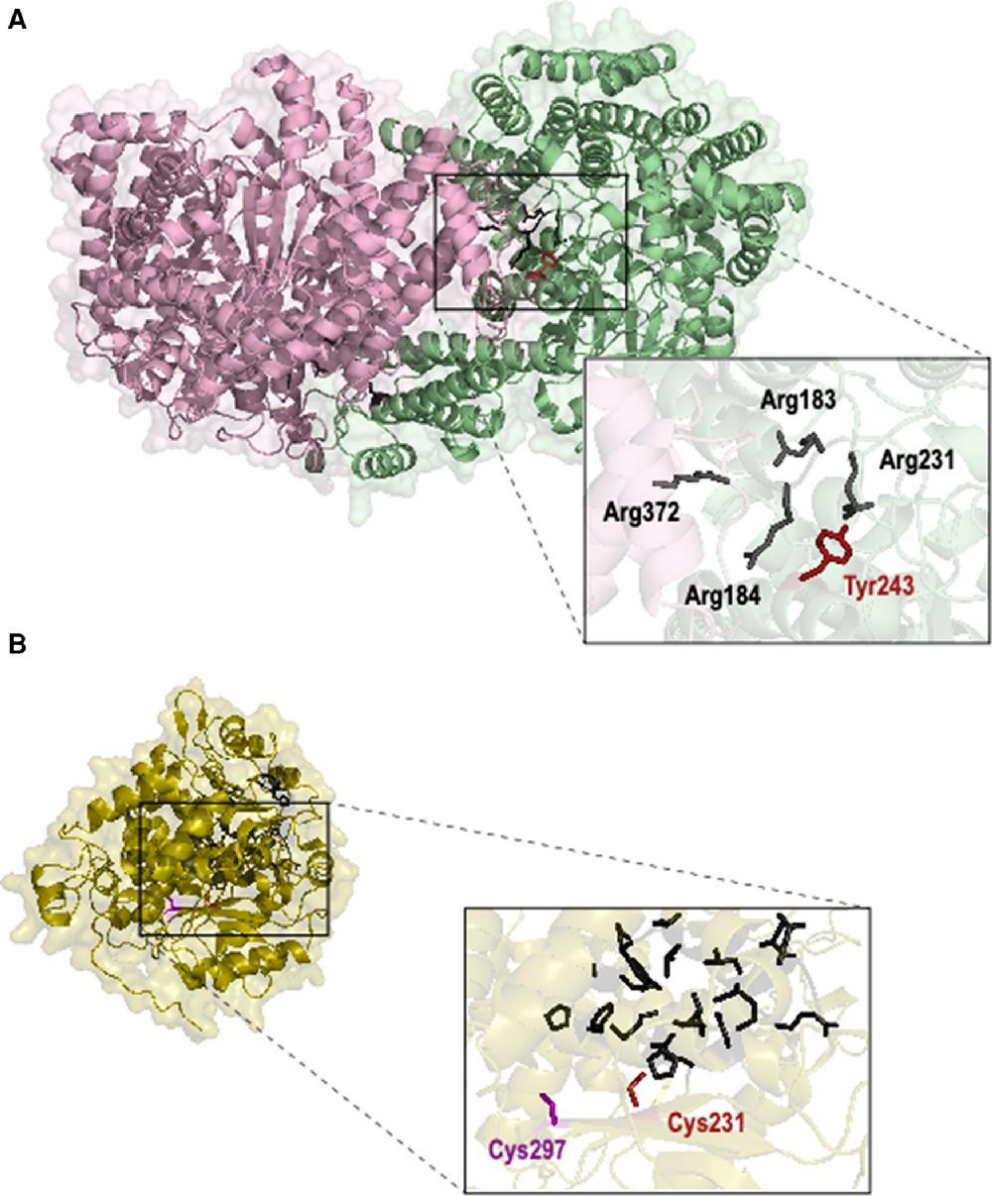
Homology modelling of *Neurachne* PEPC and NADP-ME 3D structure. Maize (*Z. mays*) PEPC and NADP-ME sequences were used as templates (see Materials and methods). Residues inferred to be under positive selection in C_4_*Neurachne* species that could significantly alter the properties of the proteins are highlighted. **A)** Schematic of C_4_*N. muelleri* PEPC. Only monomers A (pink) and B (green) are shown. Inset shows Tyr243 (red) that was inferred to be positively selected in C_4_*Neurachne* and is located next to Arg183, Arg184, Arg231, and Arg372 (dark gray) that are involved in glucose-6-phosphate binding (binding site formed by Arg372 of monomer A and Arg183, Arg184, and Arg231 of monomer B). **B)** Schematic of C_4_*N. muelleri* NADP-ME. Only monomer B (olive) is shown. Inset shows Cys231 (red) that was inferred to be positively selected in C_4_*Neurachne* and predicted to form a disulfide bond with Cys297 (purple; *N. muelleri* numbering) in the immediate vicinity of active site residues (black). When not specified, residue numbering is based on maize PEPC (sequence ID: GRMZM2G083841) and NADP-ME (sequence ID: GRMZM2G085019).

The high sequence identity shared between the C_4_-associated PEPC isoforms from maize and *Neurachne* allowed 3D structure homology modelling. This analysis indicated that Tyr243 is in close proximity to four conserved Arg residues (Fig. 9A) that are involved in the binding of the allosteric activator glucose-6-phosphate (G6P) in maize (Matsumura et al. 2002; Mancera and Carrington 2005; Takahashi-Terada et al. 2005; Yuan et al. 2006; Muñoz-Clares et al. 2020).

Positive selection analysis of the 663 aligned sites for NADP-ME homologs showed 20 sites were positively selected with PP ≥ 0.9 (3 sites with 0.9 ≤ PP < 0.95, 15 sites with 0.95 ≤ PP < 0.99, 1 site with 0.99 ≤ PP ≤ 1, 1 site with PP = 1; Fig. 8; Supplementary Table S8). The amino acid residue at position 231 (maize numbering) is the only site identified at which the residue is the same in all the C_4_ species included in the analysis (Fig. 8). Except at position 85, the NADP-ME from C_2_*N. minor* contains the same amino acid residues as the other non-C_4_ Neurachninae homologs at the identified positions (Fig. 8; Supplementary Table S8).

As for PEPC, homology modelling of the 3D structure of *Neurachne* NADP-ME was done using the maize homolog due to their shared high amino acid sequence identity and similarity (≥86% and ≥92%, respectively; Fig. 9B). A Cys residue found at position 255 of the C_4_*N. munroi* protein and at position 258 in the C_4_*N. muelleri* homolog (position 231 in maize; Fig. 8) was further investigated. While the NADP-ME–type C_4_ grasses *S. italica*, sorghum, and maize also have a Cys residue at the corresponding position, non-C_4_ species of Neurachninae contain a Val residue (Fig. 8). A Cys at this position is interesting as the side chain of the residue is very close to the active site of the protein (Fig. 9B). In C_4_*Neurachne*, a disulfide bond was predicted to form between *N. munroi* Cys255 and Cys294 (*N. munroi* numbering) and between Cys258 and Cys297 in *N. muelleri* (*N. muelleri* numbering; Supplementary Table S9).

## Discussion

In this study, 11,580 transcripts from each of seven species in the grass genus *Neurachne* were quantitatively and qualitatively analyzed. *Neurachne* is unique to our understanding of the molecular evolution of C_4_ photosynthesis as it is the only known grass group that contains distinct, closely related C_3_, C_3_-C_4_, and C_4_ species, including species representing the proposed earliest steps toward a C_4_ syndrome from the ancestral C_3_ state (Khoshravesh et al. 2020b). This study has given insights into the molecular phylogeny of this important group and revealed the gene expression patterns of closely related monocot species using different photosynthetic biochemistries. This phylogenetic, comparative approach has expanded our knowledge of the molecular mechanisms used to evolve C_4_ syndromes.

### Reconstruction of relationships within *Neurachne* using phylotranscriptomics

The phylogenetic relationships among members of the Neurachninae inferred in the current study support the earlier suggestion that C_4_ photosynthesis independently evolved in *N. muelleri* and *N. munroi* (Fig. 1; Christin et al. 2012b). In the current study, *N. muelleri* is supported as sister to the group comprising *N. alopecuroidea* (C_3_) and *N. lanigera* (C_2_-like). The phylogenetic position of *N. munroi* remained uncertain in the study of Christin et al. (2012b), which may have been due to the limited number of informative characters in the phylogenetic markers used (5 plastid and 3 nuclear markers). In the current study, *N. munroi* could be placed sister to proto-Kranz *N. annularis*, with this group in a position sister to all other *Neurachne* species. However, by looking at the proportions of gene trees and alignment sites that support this particular topology (Fig. 1), two others had relatively high support: the relationships found by Christin et al. (2012b), where *N. annularis* is sister to all other *Neurachne* species, and a topology where *N. munroi* is in this position within *Neurachne* (Supplementary Table S4). Furthermore, with regard to the phylogenetic positions of *N. minor* and *N. tenuifolia*, while PhyloNet and RAxML supported the findings of Christin et al. (2012b) with *N. minor* and *N. tenuifolia* being in a sister group relationship (though with low support), the species tree inferred with ASTRAL placed *N. tenuifolia* as sister to the group comprising *N. alopecuroidea*, *N. lanigera*, *N. minor*, and *N. muelleri* (again with low support), and within this group *N. minor* sister to the other three species (Fig. 1). Gene concordance factors of both topologies were very low (gCF = 17.5 and 20.1; Supplementary Table S4) and site concordance factors resembled each other (sCF between 33.2 and 35.9; Supplementary Table S4). Unlike the previous *Neurachne* phylogenetic reconstruction (Christin et al. 2012b), which included a limited number of informative characters, the vast amount of high-quality, independent phylogenetic markers and different phylogenetic methods applied in the current study are unlikely to be the reason for low branch support. The incongruences among the three phylogenetic methods regarding the positions of *N. minor* and *N. tenuifolia* could be caused by hybridization events within the genus *Neurachne* or incomplete lineage sorting. One of the main topologies of the current study (ASTRAL, Fig. 1) was inferred by a recent phylogenomic study of over 1,000 species of green plants (Viridiplantae; One Thousand Plant Transcriptomes Initiative 2019), including the seven *Neurachne* species analyzed in the current work. However, the One Thousand Plant Transcriptomes Initiative (2019) used a supermatrix approach for phylogenetic inference; consequently, it remains uncertain how many of the gene trees would support the main topology that was reported and how many would support alternative topologies such as those found in the current study. To further resolve the phylogenetic relationships in *Neurachne*, a wider sampling of individuals will be necessary, preferably including individuals that span the distribution ranges of each species.

### Differences in transcript profiles between *Neurachne* species

Overall, the *Neurachne* transcript investment results were reflected in the PCA done using all the *Neurachne* leaf transcript profiles. Components 2 and 3 explained nearly 29% of the variation among the species and separated them based on their photosynthetic type (Supplementary Fig. S2B), which is consistent with a PCA of *Flaveria* C_3_, C_3_-C_4_, and C_4_ species leaf transcriptome profiles (Mallmann et al. 2014).

Transcript investment in the MapMan4 category “photosynthesis” was high as expected for all *Neurachne* species (Fig. 3; Supplementary Table S2). Of the seven species examined, the two C_3_ species, *N. alopecuroidea* and *N. tenuifolia*, showed the highest investment in this category due to increases in transcripts encoding multiple components of the light reactions, including light harvesting and electron transport, as well as mRNAs coding for enzymes of the CBB cycle (Supplementary Table S2). These two species, along with proto-Kranz *N. annularis*, C_2_*N. minor* with its photorespiratory CO_2_ pump, and *N. lanigera* with its incipient C_2_ cycle (Khoshravesh et al. 2020b), also showed higher transcript investment in the category “photorespiration” than *N. munroi* and *N. muelleri* with their full C_4_ CCM (Figs. 3 and 4B; Supplementary Table S2). The relative abundance of transcripts encoding most photorespiratory proteins was highest in C_2_*N. minor* (Fig. 4B; Supplementary Table S2), a result in line with that found for C_3_-C_4_ intermediate species in the NADP-ME–type eudicot genus *Flaveria* (Mallmann et al. 2014; Lyu et al. 2021). The similar observations in eudicot *Flaveria* and monocot *Neurachne* suggest that the transcriptome profiles are characteristic of C_2_ species. Levels of several of these transcripts were also high in *N. tenuifolia*, which not only displays C_3_ leaf anatomy (Hattersley et al. 1982, 1986; Brown and Hattersley 1989; Khoshravesh et al. 2020b), but also *δ*^13^C values (Hattersley and Roksandic 1983; Moore and Edwards 1989; Christin et al. 2012b), Rubisco and PEPC activities (Hattersley et al. 1984; Hattersley and Stone 1986), gas exchange profiles (Hattersley et al. 1984, 1986), and ^14^CO_2_ metabolism (Moore and Edwards 1989) characteristic of C_3_ species. The higher transcript investment in the categories “photosynthesis” and “photorespiration” in *N*. *tenuifolia* compared with the other *Neurachne* species in the study, including its C_3_ congener *N. alopecuroidea* (Fig. 3), may represent the adaptation of a C_3_ species to an environment in which photorespiratory fluxes are high and shows that this leaf transcriptome profile is associated with C_2_ but not necessarily diagnostic for C_2_.

Also as predicted, transcript investment and the relative abundance of transcripts encoding enzymes involved in a NADP-ME–type C_4_ acid cycle and the known associated transporters were high in *N. munroi* and *N. muelleri* compared with non-C_4_*Neurachne* species (Figs. 3 and 4A; Supplementary Table S2). The relative levels of transcripts coding for several C_4_ acid cycle enzymes and transporters in leaves of C_2_*N. minor* were intermediate to those of other non-C_4_ and full C_4_*Neurachne* species (Fig. 4A), which is similar to the results seen for *Flaveria* C_3_-C_4_ intermediate species (Gowik et al. 2011; Mallmann et al. 2014, Lyu et al. 2021) and consistent with previous reports that showed *N. minor* has limited C_4_ acid cycle activity (Hattersley and Stone 1986; Hattersley et al. 1986; Moore and Edwards 1989). The significant increase in transcripts encoding the C_4_ cycle Ala-AT, AMK, BASS2, NADP-ME, PEPC, and PPDK in *N. minor* relative to C_3_, proto-Kranz, and C_2_-like *Neurachne* species (Fig. 4A; Supplementary Table S2) and higher levels of transcripts coding for proteins associated with photorespiration in this C_2_ species (Fig. 4B; Supplementary Table S2) are consistent with results in *Flaveria* and modelling studies of Mallmann et al. (2014) and Blätke and Bräutigam (2019) that support C_2_ photosynthesis as an evolutionary predecessor to a full C_4_ syndrome and the parallel operation of the two pathways in C_2_ species, facilitating amino acid recycling and N-balance between M and VS cells (Mallmann et al. 2014). Also consistent with our transcript-level results are the higher NADP-ME, PEPC, and PPDK enzyme activities in *N*. *minor* than in C_3_*Neurachne* species reported by Hattersley and Stone (1986).

Relatively moderate-to-high levels of transcripts encoding NAD-ME and/or PEP carboxykinase (PCK) were detected in leaves of all *Neurachne* species (Fig. 4A; Supplementary Table S2). Hattersley and Stone (1986) showed all the *Neurachne* species included in their study, regardless of photosynthetic types, had leaf NAD-ME activity; however, no PCK activity was detected. Adachi et al. (2023) recently noted moderate NAD-ME activity in leaves of C_3_ and C_2_*Flaveria* species and suggested that constitutive activity of a bundle sheath NAD-ME may support the decarboxylation of C_4_ acids and return of photorespiratory nitrogen (N) to M cells, activity that was replaced as a full NADP-ME–type C_4_ pathway evolved. A survey of C_4_-associated and photorespiratory enzyme abundances and activities in the leaves of a broader range of *Neurachne* species will allow further understanding of the evolution of the C_2_ and C_4_ pathways in this important grass group and this work is currently underway.

Of the two C_4_ species, *N. muelleri* had greater transcript investment than *N. munroi* in both “photosynthesis” (Fig. 3; Supplementary Table S2) and “C_4_” (Figs. 3 and 4A; Supplementary Table S2). Transcripts in “photosynthesis” that showed at least a doubling (average TPM) in *N. muelleri* relative to *N. munroi* include those coding for components of light harvesting, linear and cyclic electron transport, photophosphorylation, and the CBB cycle (Supplementary Table S2). Transcripts encoding all the enzymes in the NADP-ME–type C_4_ acid cycle and associated transport proteins are more abundant in *N. muelleri* than *N. munroi* leaves (Fig. 4A; Supplementary Table S2). Moreover, compared with the leaves of all other *Neurachne* species, those of *N. muelleri* exhibited enhanced transcript investment in the MapMan4 category “nutrient uptake” (Fig. 3). This difference was largely due to increases in abundances of transcripts encoding homologs of a Cu chaperone (CCH; Supplementary Table S2 and Supplementary Fig. S3) and adenosine 5′-phosphosulfate reductase (APR; Supplementary Table S2 and Fig. S3). More modest increases in mRNAs encoding two ATP sulfurylase isoforms (APS2 and APS3), and homologs of cystathionine γ-synthase (CGS), and a sulfate transporter (SULTR1; 3) were also found (Supplementary Table S2 and Fig. S3). In Arabidopsis, CCH appears to be involved in Cu homeostasis, including its long-distance transport and redistribution from senescing tissues to developing organs (Mira et al. 2001; Puig et al. 2007; Hoppen and Groth 2020). The first two steps in sulfate assimilation are catalyzed by APS and APR, while CGS catalyzes the first step in the synthesis of Met from Cys (Takahashi et al. 2011). In Arabidopsis, SULTR1; 3 is involved in phloem loading of sulfate for source-to-sink translocation of S (Yoshimoto et al. 2003). Interestingly, an examination of the *N. muelleri* and *N. munroi* leaf transcriptomes for mRNAs encoding proteins involved in N assimilation found several were also more abundant in *N. muelleri* relative to *N. munroi* (Fig. 4; Supplementary Table S2 and Fig. S3). These included transcripts coding for homologs of cytosolic and plastidic glutamine synthetases (GS1 and GS2), nitrate reductase (NR), nitrite reductase (NiR), and glutamate dehydrogenase (GDH). As described above, while all *Neurachne* are perennial tufted grasses (Blake 1972; Macfarlane 2007), *N. muelleri* propagates rapidly via stolons (Prendergast and Hattersley 1985). It is likely the increased investment in transcripts encoding components of C, N, S, and Cu metabolism in *N. muelleri* allows the metabolic demands of this prolific stoloniferous growth habit to be met.

C_4_ photosynthesis in species demonstrating Kranz leaf anatomy requires a high level of inter- and intracellular transport activity because of the spatial separation of primary carboxylation by PEPC in M cells and decarboxylation by NADP-ME and subsequent CBB cycle activity in VS cell chloroplasts (Weber and von Caemmerer 2010; Weber and Bräutigam 2013). Concomitantly, a high abundance of transport proteins is necessary in the membranes of chloroplasts and/or mitochondria of C_4_ species to compensate for the low turnover numbers of most transporters (Weber and von Caemmerer 2010). While the directionality and consequences of C_4_ metabolite transport between M and VS cells, as well as across organelle membranes within the two cell types have been well studied, the identities of a number of the proteins involved are not known (Majeran and van Wijk 2009; Weber and von Caemmerer 2010; Weber and Bräutigam 2013). Examination of C_4_*Neurachne* leaf transcripts exhibiting co-expression patterns identified two potential transport-associated proteins, MEP2 and TIC21 (Fig. 5). Transcripts encoding these proteins were not only highly abundant in C_4_*Neurachne* leaves, and in the case of MEP2 also in C_2_*N. minor*, but also in leaves of C_4_ species of *Flaveria* (Kümpers et al. 2017) and Salsoleae (Lauterbach et al. 2017b) compared to their C_3_ congeners. These transcripts also showed higher levels in leaves of C_4_*Salsola soda* (Salsoleae, Chenopodiaceae) when compared to cotyledons of this species, which conduct C_3_ photosynthesis (Lauterbach et al. 2017a).

Using proteomics, Bräutigam et al. (2008) found MEP2 to be highly abundant in chloroplast envelopes of maize M cells, and the same finding was independently reported by Majeran et al. (2008). Consequently, it was hypothesized that MEP2 could be either directly involved in the C_4_ cycle, for example, shuttling C_4_ cycle intermediates, or enabling increased metabolite fluxes due to C_4_ cycle activity (Bräutigam et al. 2008; Majeran and van Wijk 2009; Majeran et al. 2010; Weber and von Caemmerer 2010; Schlüter et al. 2016).

The other putative C_4_-related transport protein identified in this study due to the co-expression pattern of its cognate transcripts, TIC21 (Fig. 5), was predicted to localize to the inner membrane of M cell chloroplasts in maize (Majeran et al. 2008). Based on protein analyses (Kikuchi et al. 2009) and Arabidopsis *tic21* (*cia5*) mutants exhibiting a pale phenotype and accumulation of unprocessed chloroplast precursor proteins (Teng et al. 2006), it has been proposed that TIC21 (CIA5) is part of the import channel in the inner chloroplast membrane. However, in another study, TIC21 (PIC1) was characterized as an iron transporter, as Arabidopsis *pic1* mutants had impaired chloroplast development, but protein import into the chloroplast seemed to be unaffected (Duy et al. 2007). More recently, a dual function in chloroplast protein import and iron transport across the chloroplast envelope has been proposed for this protein (Paila et al. 2015).

The findings that transcripts encoding MEP2 and TIC21 are more abundant in the leaves of C_4_ than C_3_ species (this study; Kümpers et al. 2017; Lauterbach et al. 2017b) together with reports stating that protein abundance of both were higher in M than in bundle sheath tissue of maize (Bräutigam et al. 2008; Majeran et al. 2008), emphasize their potential significance in C_4_ photosynthesis and warrant their further characterization.

### Different splice forms of the *β*-CA1 primary transcript were co-opted for *Neurachne* C_4_ syndromes

Both C_3_ and C_4_ plants contain cytosolic and chloroplastic isoforms of *β*-CA, with most of the *β*-CA activity in C_4_ plants localizing to the M cell cytosol where it catalyzes the first step in the C_4_ pathway, providing bicarbonate for PEPC (Hatch and Burnell 1990). By contrast, most *β*-CA activity in C_3_ plants is found in M cell chloroplasts (Everson and Slack 1968) where its role remains inconclusive (DiMario et al. 2017; Rudenko et al. 2021). Two genes encode *β*-CA in *Neurachne*, *β*-*ca1* and *β*-*ca2*, with both producing primary transcripts that are alternatively spliced (Clayton et al. 2017). Results presented here from leaf transcriptomes (Fig. 4) and RT-qPCR (Fig. 6) analyses and by Clayton et al. (2017) indicate, for all *Neurachne* species examined thus far, levels of the mature transcripts encoding cytosolic *β*-CA2a and chloroplastic *β*-CA2b are relatively low (Fig. 6; Clayton et al. 2017) and, as such, likely encode aneupleurotic forms of the enzyme (DiMario et al. 2017). By contrast, these analyses also show *β*-CA1 mRNAs are abundant transcripts in the leaves of all *Neurachne* species. In C_4_*N. munroi*, as well as C_3_*N. alopecuroidea* and C_2_*N. minor*, *β*-CA1a transcripts are the most abundant form of mRNA encoding *β*-CA, while in the leaf tissue of C_4_*N. muelleri*, *β*-CA1b transcripts are the most abundant (Fig. 6; Clayton et al. 2017). In all examined *Neurachne* species, *β*-CA1b is a cytosolic enzyme (Fig. 7; Clayton et al. 2017), whereas *β*-CA1a is a chloroplastic isoform except in *N. munroi* where it has been proposed that the loss of the sequence encoding the chloroplast transit peptide allowed co-option of *β*-CA1a into the C_4_ pathway of this species (Clayton et al. 2017). A similar mechanism has been reported for the evolution of the C_4_-associated *β*-CA in the eudicot *F. bidentis* (Tanz et al. 2009). Consequently, our results suggest that the two evolutionary origins of C_4_ photosynthesis in *Neurachne* were enabled by different genetic mechanisms: in *N. muelleri*, the splice form encoding the ancestral C_3_ cytosolic *β*-CA1b isoform was co-opted, while in *N. munroi* the loss of the transit peptide from the splice form encoding the ancestral C_3_ chloroplastic *β*-CA1a gave rise to its C_4_-associated *β*-CA. This underscores the most parsimonious explanation for the observed phylogenetic relationships and trait distributions in the Neurachninae is the independent evolution of C_4_ photosynthesis on two distinct branches.

### The N-terminal region of C_4_*Neurachne* PEPC may be involved in allosteric regulation of the enzyme

Holoenzymes of C_4_-associated PEPC that catalyze the carboxylation of PEP in the M cytosol are homotetramers that are described as a “dimer of dimers” (Kai et al. 1999; Matsumura et al. 2002). In C_3_ species, PEPC homologs also function as tetramers; however, in these species, the enzymes are involved in a number of nonphotosynthetic roles such as seed development and germination, fruit ripening, and stomatal opening (Chollet et al. 1996; Lepiniec et al. 2003; O’Leary et al. 2011). While PEPC homologs from C_3_ and C_4_ species share high sequence identity, major differences exist in their kinetics, regulatory properties, expression levels, and spatial expression (Bauwe and Chollet 1986; Ernst and Westhoff 1997; Svensson et al. 1997, 2003; Bläsing et al. 2000; Gowik and Westhoff 2011; Langdale 2011; DiMario and Cousins 2019; DiMario et al. 2021, 2023). Some of these differences evolved through changes in regulatory elements and their associated transcription factors (Hibberd and Covshoff 2010; Górska et al. 2019, 2021; Gupta et al. 2020); however, the high sequence conservation between PEPC homologs from C_3_ and C_4_ species suggests that only a few amino acid changes were required to evolve a C_4_-associated PEPC from an ancestral C_3_ isoform. Indeed, a single amino acid exchange, Ala (C_3_) to Ser (C_4_) close to the active site in the C-terminus (amino acid position 780; maize numbering) was shown to make the C_3_*Flaveria pringlei* PEPC isoform more C_4_-like by increasing its K_PEP_ (Bläsing et al. 2000) and, more recently, to play a role in balancing *K_PEP_* and *K_HCO3_* (DiMario and Cousins 2019). In nearly all plant lineages investigated so far, including *Neurachne*, PEPC homologs from C_4_ species contain a Ser residue at the position corresponding to 780 of the maize C_4_-associated PEPC, while homologs from non-C_4_ species have Ala (Fig. 8; Bläsing et al. 2000; Gowik et al. 2006; Lara et al. 2006; Christin et al. 2007, 2011, 2012a, 2012b; Besnard et al. 2009; Christin and Besnard 2009; Rosnow et al. 2014; Khoshravesh et al. 2020a; Moody et al. 2020; Yamamoto et al. 2022; DiMario et al. 2023).

In addition to Ser780, other residues in the C-terminal region of C_4_-associated PEPC have a role in the kinetic properties of the enzyme. For example, DiMario et al. (2021) reported that residues 528 to 880 are involved in the *K_HCO3_* of PEPC isoforms in C_4_ grasses; however, the exact amino acid residues responsible were not identified. At the C-terminal end of the C_4_-associated PEPC isoform in *Flaveria*, Paulus et al. (2013) showed the replacement of Arg with a Gly residue at position 890 (maize numbering) is important for decreased malate inhibition and, based on amino acid sequence identity, potentially has the same role in PEPC homologs of the Andropogoneae grasses maize, sugarcane (*Saccharum officinarum*), and sorghum. Yet, other C_4_ grasses, including *N*. *munroi* and *N*. *muelleri*, contain C_4_-associated PEPC isoforms with an Arg residue at position 890 (DiMario et al. 2023), which suggests different evolutionary mechanisms were responsible for malate tolerance in these species.

A previous positive selection analysis using *Neurachne* genomic sequences encoding the C-terminal regions of PEPC found eight amino acid positions showing C_4_-specific positive selection (Christin et al. 2012b). Three of these positions (531, 780, and 794; maize numbering) were identified as positively selected in the current study using *Neurachne* leaf transcriptome sequences (Fig. 8; Supplementary Table S8). A potential explanation for this apparent discrepancy is that most *Neurachne* species are autopolyploids with intraspecific polyploidy common (Christin et al. 2012b). This along with limited population sampling could account for at least some of the difference between the two *Neurachne* studies. For position 531, a Pro residue was found for all C_4_-associated PEPC homologs included in the current study, while homologs of the non-C_4_ species contain an Ala (Fig. 8). This same substitution was previously found for other grass and some eudicot C_4_ lineages (Christin et al. 2007; Christin and Besnard 2009). By contrast, at position 794, non-polar amino acid residues are found for all species with no residue consistency among the C_4_ species (Fig. 8), which also agrees with results of other positive selection analyses (Christin et al. 2007, 2011, 2012b; Rosnow et al. 2014; Khoshravesh et al. 2020a).

In the current study, positive selection was also found for *Neurachne* C_4_ PEPC at position 572 where a Gln residue is substituted for Glu in the non-C_4_ homologs (Fig. 8; Supplementary Table S8). The other C_4_ PEPC homologs included in the current study also contain Gln at position 572 (Fig. 8), and the same substitution was found in previous positive selection analyses examining other monocot and eudicot families containing C_4_ members (Besnard et al. 2009; Christin and Besnard 2009; Christin et al. 2011, Khoshravesh et al. 2020a). Two further sites, 668 and 836 (Fig. 8), which had not been reported in previous positive selection analyses, were detected in the C-terminal region of the C_4_*Neurachne* enzymes. Both C_4_*Neurachne* homologs contain an Ile residue at position 668 and an Ala at 836; however, these residues are not consistent at the corresponding position of the other C_4_ PEPC homologs included in the study (Fig. 8). The effects of these C-terminal substitutions in C_4_*Neurachne* PEPC on the kinetics of the enzymes relative to the homologs of non-C_4_*Neurachne* congeners await further analyses.

The N-terminal regions of C_4_-associated PEPC isoforms have been largely overlooked with respect to positive selection analyses and C_4_ enzyme function. Svensson et al. (1997) showed that in *Flaveria*, this region is important for activation of the enzyme via the allosteric effector G6P, and seven residues at positions 226 to 232 in the N-terminal region of the maize C_4_-associated PEPC appear to be key for the kinetics and regulation of the enzyme (Yuan et al. 2006). More recently, Rosnow et al. (2015) found positions 364 and 368 to be under positive selection in C_4_*Suaeda* species and proposed involvement of these residues in substrate and allosteric effector binding. In the current study, by including the complete coding sequences of the *Neurachne* PEPC isoforms, positions 243 and 320 were identified to be under positive selection in C_4_*Neurachne* (Fig. 8). Recently, Yamamoto et al. (2022) also noted position 243 as a positively selected site in the C_4_-associated PEPC isoforms included in their analyses.

Amino acid residues with alternative nonpolar side chains account for the differences at position 320 of the C_4_ and non-C_4_ PEPC homologs included in the current study (Fig. 8). However, positive selection at position 243 (242 *Neurachne* numbering) in C_4_*Neurachne* PEPC is conspicuous in that this site is occupied by a Tyr (uncharged polar) residue, while the corresponding position in the PEPC homologs of non-C_4_ Neurachninae congeners contains nonpolar Gly or Ala residues (Fig. 8; Supplementary Table S8). Similarly, the PEPC isoforms from the NADP-ME-type C_4_ grasses maize, sorghum and *S. italica* have a Tyr residue at the corresponding position (Fig. 8). Furthermore, a Tyr residue is present at the homologous position in PEPC isoforms from C_4_*Hammada scoparia* and C_4_*Salsola oppositifolia*, while again Ala is found in the homolog from C_3_*Salsola webbii* (Lauterbach et al. 2017b, Supplementary data). By contrast, an Ala residue is found at the corresponding position in PEPC isoforms from C_4_ and non-C_4_*Flaveria* species (Engelmann et al. 2003).

Homology modelling of the quaternary structure of C_4_*Neurachne* PEPC predicts Tyr242 is located within an alpha helix near four Arg residues (Fig. 9A) that contribute to binding of the allosteric activator G6P (Matsumura et al. 2002; Mancera and Carrington 2005; Takahashi-Terada et al. 2005; Yuan et al. 2006; Muñoz-Clares et al. 2020). The substitution of Tyr with its phenolic OH-group for non-polar Gly and Ala in some C_4_ lineages may influence G6P binding and allosteric regulation of PEPC in these taxa. This selection may have been important in C_4_ evolution and/or establishment of the C_4_ pathway in monocots and some eudicot lineages, for example, chenopods, while in *Flaveria*, a different site/region led to higher G6P activation of the C_4_-associated PEPC.

The diversity in amino acids at what have been identified as positively selected or C_4_-specific sites in PEPC isoforms of different taxa may reflect the PEPC homolog co-opted or local selection pressures such as environmental growth conditions in different lineages during C_4_ evolution. In *Neurachne*, 96% amino acid sequence identity is shared between the PEPC homologs of the two C_4_ species, including nearly all the positively selected sites identified in this study, and this may reflect a lateral gene transfer occurred during the evolution of C_4_ photosynthesis in these species (Christin et al. 2012b). Such an event is supported by the observation that both coding and intron regions of *N. munroi* and *N. muelleri* PEPC exhibit nucleotide sequence homology (Christin et al. 2012b); however, with the available data, the direction of transfer, *N. munroi* to *N. muelleri* or vice versa, cannot be determined. Lateral gene transfer events supporting the optimization of C_4_ photosynthesis have also been reported among *Alloteropsis* species (Christin et al. 2012a).

*Neurachne* with its distinct, closely related species using different photosynthetic biochemistries offers a unique monocot system by which the diversity in amino acid residues and the residues enabling a C_4_-type PEPC can be tested through expression and kinetic characterization of recombinant proteins with targeted amino acid exchanges. Experiments are now underway to examine the effects of both N- and C-terminal positively selected sites on the kinetic properties of *Neurachne* PEPC isoforms.

### A valine-to-cysteine exchange in NADP-ME may be important for gaining C_4_ properties in *Neurachne*

The decarboxylation of C_4_ acids in VS tissue is a significant feature of the C_4_ CCM as it raises the concentration of CO_2_ around Rubisco, which is found only in the chloroplasts of these cells (Huber et al. 1976), and consequently greatly reduces the oxygenase activity of the enzyme and photorespiration. Previous work on the C_4_-associated NADP-ME from grasses showed the enzyme demonstrates high activity as a homotetramer at pH 8.0 (Maier et al. 2011; Alvarez and Maurino 2023). At pH 7.0, the enzyme loses its active quaternary state and is allosterically inhibited by its substrate, malate (Maier et al. 2011; Alvarez and Maurino 2023). Moreover, the activity of the maize (Alvarez et al. 2012) and sorghum (Saigo et al. 2013) C_4_-associated NADP-ME has been shown to be redox regulated in vitro. These C_4_-specific properties did not evolve via a single mutation in the ancestral C_3_ NADP-ME gene, but instead through a combination of several key modifications that differed depending on the monocot lineage (Christin et al. 2009; Alvarez et al. 2013, 2019; Alvarez and Maurino 2023).

In a previous study, Christin et al. (2009) highlighted seven amino acids in NADP-ME isoforms being under positive selection in C_4_ grasses. Of these, the residue at position 231 (maize numbering) was also found in the current study to be under positive selection in the two C_4_*Neurachne* species (Fig. 8). In C_4_*Neurachne* NADP-ME homologs, as in those from other NADP-ME-type C_4_ grasses, a Cys residue is found at position 231, while in the homologs of related non-C_4_ species, the position contains a Val (Fig. 8; Supplementary Table S8; Christin et al. 2009). At the corresponding position, the C_4_ eudicots *Hammada scoparia*, *Salsola oppositifolia*, and *Salsola soda* also contain a Cys residue, while C_3_*Salsola webbii* has a Val (Lauterbach et al. 2017b, Supplementary data). Depending on the local redox status, two Cys residues may form a disulfide bond that leads to protein structural changes, potentially modifying function. As noted above, in NADP-ME–type C_4_ species, NADP-ME is located in the VS chloroplasts where it decarboxylates the malate produced in M cells and may be under redox control (Alvarez et al. 2012; Saigo et al. 2013). In maize, Cys231 is located in the immediate vicinity of the active site and was one of four Cys residues predicted to form disulfide bonds based on the structure of the human mitochondrial NAD(P)-ME (Alvarez et al. 2012). In the current study, the recently published crystal structure of the C_4_-associated NADP-ME from maize (Alvarez et al. 2019) was used to investigate the potential of the Cys residues in *Neurachne* NADP-ME to form disulfide bonds. This analysis indicated that the Cys residues in C_4_*Neurachne* NADP-ME that correspond to maize Cys231, i.e. Cys255 in *N. munroi* and Cys258 in *N. muelleri*, are predicted to form disulfide bonds with Cys294 and Cys297, respectively (Supplementary Table S9; Fig. 9B), thereby potentially changing the catalytic activity and/or conformation of the enzymes. The current study also predicted maize Cys231 to form a disulfide bond with Cys192 (Supplementary Table S9). Alvarez et al. (2012) showed that replacing Cys231 with an Ala residue in maize C_4_-associated NADP-ME led to a lower catalytic efficiency for both NADP and malate relative to the wild-type enzyme; however, Cys231 did not appear to be involved in redox regulation of the enzyme in vitro. Instead, in experiments using recombinant maize NADP-ME, Cys246 was implicated in redox regulation of the enzyme (Alvarez et al. 2012). In all *Neurachne* NADP-ME homologs, a Ser residue is at the position corresponding to maize Cys246, while in *S. italica*, it is an Ala residue (Supplementary Fig. S6). It is interesting to note that in the current study, Cys246 in maize and sorghum is predicted to form a disulfide bond with Cys270, which corresponds to *N. munroi* Cys294 and *N. muelleri* Cys297 (Supplementary Table S9).

The independent evolution of Cys231 in multiple NADP-ME–type C_4_ species, both grasses and Salsoleae (Fig. 8; Christin et al. 2009; Lauterbach et al. 2017b), together with the observed changes in substrate affinity and kinetics in site-directed Cys231Ala mutants (Alvarez et al. 2012), clearly indicate that Cys231 is important in the adaptation of C_4_-associated NADP-ME isoforms. Further studies are needed to determine if the Cys residues near the NADP-ME active site in multiple C_4_ lineages form disulfide bonds and, if so, whether they play a role in the kinetics and/or redox regulation of the enzymes.

Based on the cleavage site of the maize C_4_-associated NADP-ME chloroplast transit peptide being in the region between amino acid residues 61 and 66 (Maurino et al. 1996; Saigo et al. 2013), 14 additional positively selected sites were predicted in the mature C_4_*Neurachne* NADP-ME homologs (Fig. 8; Supplementary Table S8). None of these sites was detected in a previous positive selection analysis (Christin et al. 2009). Interestingly, for each of these sites, the same amino acid residue was found in the corresponding positions of the two C_4_*Neurachne* homologs; however, none of the sites showed complete amino acid conservation in the other C_4_ NADP-ME homologs included in the current study, nor were any of the residues conserved in the C_2_*N. minor* protein (Fig. 8).

Previous site-directed mutagenesis work with the NADP-ME from NADP-ME–type grasses identified three amino acid residues (Glu339, Gln503, and Leu544), in addition to Cys246, that appear to be important for the functioning of the C_4_-associated isoforms in these species (Alvarez et al. 2019, Supplementary data); however, none of these residues showed positive selection in *Neurachne* (Supplementary Fig. S6). With one exception, the amino acid residues of the *Neurachne* NADP-ME homologs are identical at each of these three positions regardless of the CO_2_ assimilation pathway used by the species (Supplementary Fig. S6). Moreover, the *Neurachne* homologs exhibit the non-C_4_ NADP-ME amino acid residues identified previously (Alvarez et al. 2019). The exception, Glu339, has been implicated in the high affinity of NADP-ME for malate and malate inhibition at low pH (Alvarez et al. 2019) and was identified in a previous positive selection analysis (Christin et al. 2009). In C_4_*N. munroi* a Glu residue is at position 339 as in maize, sorghum, and *Setaria* C_4_ NADP-ME homologs (Supplementary Fig. S6; Alvarez et al. 2019); however, C_4_*N. muelleri* and all the non-C_4_*Neurachne* homologs contain an Ala residue (Supplementary Fig. S6), as do the non-C_4_ NADP-ME isoforms examined by Alvarez et al. (2019). In addition to the above amino acid residues recognized as C_4_ adaptations, a 9 to 15 amino acid deletion near the N-terminus (DelN; Alvarez et al. 2019) and a Phe residue at position 140 of maize C_4_-associated NADP-ME have been shown to be involved in the oligomerization of the enzyme (Alvarez et al. 2019). Neither the deletion nor the substitution is seen in C_4_*Neurachne* NADP-ME. Instead, all *Neurachne* NADP-ME homologs share sequence homology with non-C_4_ NADP-ME homologs at DelN and an Ile at the position corresponding to maize Phe140 (Supplementary Fig. S6).

It has been proposed that in maize and sorghum, the gene encoding the C_4_-associated NADP-ME evolved through two gene duplication events (Tausta et al. 2002; Christin et al. 2009). The first event involved the duplication of the gene coding for an ancestral C_3_ cytosolic isoform, with one copy gaining a sequence encoding a chloroplast transit peptide and subsequently duplicated. Neofunctionalization, including bundle sheath cell-preferential expression, of one of these paralogs resulted in the isoform involved in C_4_ cycle activity, while the other encodes an isoform maintaining the ancestral C_3_ housekeeping roles and defense responses (Tausta et al. 2002). By contrast, the genomes of *S. italica* and *Setaria viridis* contain only one gene encoding a plastid NADP-ME isoform, which performs both C_4_ cycle and non-C_4_ activities (Calace et al. 2021). The *Neurachne* leaf transcriptome data also support a single gene coding for a plastidic NADP-ME isoform. While evidence suggests the C_4_-associated NADP-ME in different C_4_ NADP-ME-type lineages evolved through recurrent recruitment of the same ancestral gene, and in many cases the same amino acid residue was selected during adaptation of the enzyme (Christin et al. 2009), the diversity of other mutations indicates multiple amino acid combinations can lead to NADP-ME isoforms that efficiently support C_4_ cycle activity (Fig. 8; Christin et al. 2009; Alvarez et al. 2019; Alvarez and Maurino 2023).

## Conclusion

Comparative transcriptomics of more than 11,500 genes from seven *Neurachne* species demonstrating different photosynthetic biochemistries have permitted the robust examination of species relationships within the genus, including that of the two C_4_ species which show independent evolutionary origins. The molecular mechanism enabling the evolution of the *β*-CA that catalyzes the first step in the C_4_ pathway further supported these independent origins in that different splice forms of the *β*-*ca1* gene were co-opted to fill this role in C_4_*N. muelleri* and C_4_*N. munroi*. Transcriptional investment and mRNA abundance profiles in the categories “C_4_ photosynthesis” and “photorespiraton” reinforced the hypothesis that extant members of *Neurachne* represent steps along the C_3_ to C_4_ evolutionary continuum in the genus. Putative membrane proteins showing gene expression patterns similar to those of genes encoding C_4_-associated proteins were identified and offer an opportunity to fill a knowledge gap in the understanding of metabolite transfer in C_4_ NADP-ME-type photosynthesis. Similarly, the recognition of positively selected amino acid residues in C_4_*Neurachne* PEPC and NADP-ME homologs now enables the molecular dissection and identification of the changes that were responsible in the conversion of ancestral C_3_ homologs into C_4_-associated isoforms and indicates that while the overall evolutionary path appears similar, evolution at the single enzyme level is not always convergent. The dataset also uncovered differences in transcript abundances between C_4_*N. muelleri* and all its congeners, including the C_4_*N. munroi*, that likely explain its unique growth habit within *Neurachne*. This comparative study has established the importance and utility of *Neurachne* as a model group to understand the molecular evolution of C_4_ photosynthesis in the grasses.

## Materials and methods

### Plant material

*Neurachne alopecuroidea*, *N. annularis*, *N. lanigera*, *N. minor*, *N. muelleri*, *N. munroi*, and *N. tenuifolia* were grown in naturally illuminated glasshouses in the Plant Growth Facility at the University of Western Australia, Perth, Australia, from either seed or whole plants collected from their natural habitats (Supplementary Table S10) as described by Khoshravesh et al. (2020b). Recent fully expanded leaves of all seven species (each species with biological triplicates except *N. tenuifolia*, which due to seed shortages, had only biological duplicates) were harvested, immediately frozen in liquid nitrogen and stored at −80 °C until RNA isolation. Photosynthetic-type designation followed Khoshravesh et al. (2020b) for *Neurachne annularis* (proto-Kranz), *N. lanigera* (C_2_-like), and *N. minor* (C_2_) and Hattersley et al. (1982) for *N. alopecuroidea* (C_3_), *N. tenuifolia* (C_3_), *N. muelleri* (C_4_), and *N. munroi* (C_4_).

### RNA isolation and mRNA sequencing

Total RNA was isolated from 100 mg of ground leaf material using a phenol/chloroform method as described by Clayton et al. (2017). RNA-Seq libraries were prepared using the TruSeq RNA Sample Preparation Kit (Illumina) following the manufacturers’ protocol. Paired-end sequencing was performed using the Illumina HiSeq2000 platform at Heinrich-Heine University, Düsseldorf, Germany.

### Phylogenetic inference

To infer the molecular phylogeny of *Neurachne*, leaf transcriptome data were processed as described in Yang and Smith (2014); bitbucket.org/yanglab/phylogenomic_dataset_construction/. Paired-end sequencing reads were checked for quality using FASTQC tool (www.bioinformatics.babraham.ac.uk/projects/fastqc/) and filtered and trimmed using Trimmomatic v.0.38 (Bolger et al. 2014). To identify reads resulting from genes encoded in chloroplast and mitochondrial genomes, filtered reads were mapped against the chloroplast and mitochondrial genomes of maize (*Zea mays*; NC_001666 and NC_001400, respectively) using bowtie2 v.2.3.4.1 (Langmead and Salzberg 2012) and only reads that derived from the nuclear genome were included in downstream analyses. Reads were de novo assembled for each species separately using Trinity v.2.1.1 (Grabherr et al. 2011). Transcripts were then filtered and translated using TransRate v.1.0.3 (Smith-Unna et al. 2016), Corset v.1.05 (Davidson and Oshlack 2014), Salmon v.0.9.1 (Patro et al. 2017), and TransDecoder v.5.3.0 (transdecoder.github.io). Transcripts were reduced via clustering using CD-HIT-EST v.4.7 (Li and Godzik 2006; Fu et al. 2012), and homologs were inferred via an all-by-all BLAST v.0.6.9 search with cut-off *e*-value of 1e−10 followed by Markov clustering with filtered *e*-values from BLAST hits using MCL v.14.137 (van Dongen 2000; Enright et al. 2002). As recommended (Yang and Smith 2014), filtering in MCL was done with different values for hit fraction cut-off (0.3, 0.4, and 0.5) inflation (1.4 and 2.0), and only clusters including sequences from all eight species (seven *Neurachne* species plus the outgroup taxon *Setaria italica*) were used in subsequent analyses. For clusters with less than 1,000 sequences, each cluster was aligned using MAFFT v.7.407 with “–genafpair –maxiterate 1000” (Katoh and Standley 2013). Resulting alignments were cleaned using the pxclsq function with “-p 0.1” in Phyx (Brown et al. 2017), and trees were inferred using RAxML v.8.2.12 (Stamatakis 2014). For clusters containing 1,000 or more sequences, each cluster was aligned using PASTA v.1.8.5 (Mirarab et al. 2014, 2015), and resulting alignments cleaned using the pxclsq function with “-p 0.01” in Phyx, with trees inferred using FastTree v.2.1.10 (Price et al. 2010). After visual inspections of at least 10 resulting alignments and trees per varied parameter (i.e. hit fraction cut-off and inflation value; see above) to evaluate parameter settings, a hit fraction cut-off of 0.3 and inflation value of 1.4 were chosen and downstream analyses were based on trees resulting from these settings. Terminal branches that were longer than a length cut-off of 0.05 and more than 10 times as long as its sister (relative cut-off) or longer than 10 (absolute cut-off) were trimmed, monophyletic tips belonging to the same taxon masked, and subsequently, deep paralogs cut using a stringent cut-off of 0.1 (“long_internal_branch_cutoff”; bitbucket.org/yanglab/phylogenomic_dataset_construction/; Yang and Smith 2014). To produce the final ortholog trees, the “one-to-one/1to1” method was used, which uses only homologs that have no duplicated taxon (Yang and Smith 2014). Finally, the remaining multiple sequence alignments with trimmed and/or masked tips (see above) were used for inference of phylogenetic relationships of *Neurachne*. Phylogenetic inference was done using three alternative approaches. First, a tree was calculated using RAxML v.8.2.12 (Stamatakis 2014) with a supermatrix resulting from concatenation of 671 marker alignments (i.e. regions with ≥1,200 base pair (bp) length and present in all species). GTRGAMMA was used as the model, and partitions of the multi-gene alignment were kept, allowing estimation of individual alpha-shape parameters, GTR rates, and empirical base frequencies. Second, an individual gene tree was calculated for each marker and a species tree was calculated based on these gene trees with local PP using ASTRAL v.5.6.3 with default settings (Mirarab et al. 2014; Mirarab and Warnow 2015). For evaluation of support values found in the ASTRAL and RAxML analyses, IQ-TREE v.2.0 (Minh et al. 2020) was used for calculation of gCF and sCF, as well as gDF and sDF. While the gCF calculation was based on the gene trees obtained with RAxML, the sCF calculation was based on 1,000 quartets (“–scf 1000” option). When inferring phylogenetic relationships of closely related species, like those in *Neurachne*, a phylogenetic network can be better suited to depict the relationships than a phylogenetic tree. Consequently, in a phylogenetic network approach, the “InferNetwork_MPL” function in PhyloNet v.3.7.1 (Than et al. 2008; Yu and Nahkleh 2015; Wen et al. 2018) and the Neighbor-Net algorithm (Bryant and Moulton 2004) in SplitsTree4 (Huson and Bryant 2006) were used to infer phylogenetic networks. For visualization, FigTree v.1.4.3 (http://tree.bio.ed.ac.uk/software/figtree/) and Dendroscope v.3.5.10 (Huson and Scornavacca 2012) were used.

### De novo transcriptome assembly and analysis of differential gene expression

For each species, de novo assembly was conducted using quality-filtered reads (see above) of all replicates of the respective species with default parameters in Trinity v.2.1.1 (Grabherr et al. 2011). Quality of assemblies was assessed with BUSCO v.3.0 (Benchmarking Universal Single-Copy Orthologs) with the Liliopsida odb10 dataset (Simão et al. 2015; Kriventseva et al. 2019) and TransRate v.1.0.3 (Smith-Unna et al. 2016). The number of contigs was then reduced by clustering via CD-HIT-EST v.4.7 (Li and Godzik 2006; Fu et al. 2012). Only contigs with an open reading frame (ORF) were included in the downstream analysis, which was conducted with TransDecoder v.5.3.0 (github.com/TransDecoder/TransDecoder) followed by another round of CD-HIT-EST. Orthology assignment between the seven resulting assemblies was done by conditional reciprocal best (crb) BLAST v.0.6.9 (Aubry et al. 2014) run locally using *S. italica* v.2.2 from Phytozome (Bennetzen et al. 2012) as reference. Orthologous sequences between each of the assemblies (see above) and *S. italica* were assigned via crb-BLAST. In the downstream analyses, only contigs were included that had ortholog assignments for all seven species. Reads of each of the replicates were separately mapped against these reduced data sets via bowtie2 v.2.3.4.1 (Langmead and Salzberg 2012). Samtools v.1.3 (Li et al. 2009) was used for data re-formatting and manipulation, and extraction of read counts (excluding supplementary alignments). These read counts were used for differential gene expression analysis and pairwise comparison between all seven *Neurachne* species was statistically evaluated using edgeR (Robinson et al. 2010) classic mode in R (R Core Team 2018). Hierarchical clustering using Pearson Correlation and PCA of log_2_ transformed reads counts (transcripts per million, TPM) and *z*-scores were done with Multiexperiment Viewer (MeV) v.4.9 (http://mev.tm4.org/). Pathways were defined using MapMan4 categories (Schwacke et al. 2019) and the two additional pathways “C_4_” and “photorespiration”, and the average TPM values per species were used. Co-expressed gene clusters of all expressed transcripts were identified with Clust v.1.10.7 (Abu-Jamous and Kelly 2018).

### *N. muelleri* CA expression and localization

RNA isolation and transcript abundance of *Neurachne β*-CA using RT-qPCR assays were performed as described by Clayton et al. (2017), including the use of three biological replicates (Supplementary Table S10) and three reference genes, tests of amplification specificity and efficiency, and controls. As the *UBQ5* reference gene had the highest amplification efficiency of the three reference genes (Clayton et al. 2017), data are presented normalized to this gene. Statistical significance between transcript abundances was evaluated using Student's *t*-test, with *P* ≤ 0.05 deemed significant.

The ORFs encoding *N. muelleri β*-CA isoforms 1a, 1b, 2a, and 2b were fused to that of GFP as described by Clayton et al. (2017). Transformation of *Agrobacterium tumefaciens*, infiltration of tobacco (*Nicotiana benthamiana*) leaves, protoplast isolation, and visualization of GFP and chlorophyll autofluorescence were carried out as described previously (Clayton et al. 2017).

### Positive selection analysis of C_4_-associated proteins

To test whether positive selection affected the evolution of C_4_-associated proteins in *Neurachne*, branch-site test of *codeml* implemented in PAML v.4.9 (Yang 2007) and PAMLX v.1.3.1 (Xu and Yang 2013) was used. Since phylogenetic analysis showed some conflicts regarding the relationships of *N. tenuifolia* (see below), all positive selection analyses were conducted using the three different topologies (Supplementary Fig. S5): (i) *N. tenuifolia* sister to the group comprising *N. minor* and *N. alopecuroidea*, *N. lanigera*, and *N. muelleri*; (ii) *N. tenuifolia* in a polytomy together with *N. minor* and the group comprising *N. alopecuroidea*, *N. lanigera*, and *N. muelleri*; and (iii) *N. tenuifolia* sister to *N. minor*. Nucleic acid sequence information for the transcripts encoding the C_4_-associated Ala-AT, AMK, Asp-AT, BASS2, *β*-CA1, DiT1, NADP-MDH, NADP-ME, PEPC2, PEPC-K, pyrophosphatase (PPase), and PPDK were taken from the *Neurachne* RNA-Seq data generated in this study and from publicly available data for two *Thyridolepis* species (Matasci et al. 2014; One Thousand Plant Transcriptomes Initiative 2019; sample accession numbers SAMEA104170826 and SAMEA104170827), which were used as outgroups. The two branches leading to the C_4_ species *N. muelleri* and *N. munroi* were specified as foreground branches, and the branch-site model (model = 2, NSites = 2) was compared with the corresponding null model (model = 2, NSites = 2, fix_omega = 1, omega = 1). Significance between the alternative and null models was tested using a likelihood ratio test (df = 1; Yang and Nielsen 2002; Yang 2007). In case the likelihood ratio test showed that the alternative model was significantly better suited, sites under positive selection were identified using Bayes empirical Bayes (BEB, Yang et al. 2005).

### Homology modelling of *Neurachne* PEPC and NADP-ME

Homology modelling of the 3D structures of *Neurachne* PEPC and NADP-ME proteins was done using I-TASSER (Zhang 2008; Roy et al. 2010; Yang et al. 2015) and SWISS-MODEL (Waterhouse et al. 2018). Published protein structures of PEPC (PDB ID: 1JQO, Matsumura et al. 2002) and NADP-ME (PDB ID: 5OU5, Alvarez et al. 2019) from maize were obtained from Research Collaboratory for Structural Bioinformatics Protein Data Bank (www.rcsb.org; Berman et al. 2002; Burley et al. 2019), and used as the template in SWISS-MODEL. DIpro (Baldi et al. 2004; Cheng et al. 2006a, 2006b) was used to predict cysteine disulfide bonds within NADP-ME.

## Supplementary Material

kiae424_Supplementary_Data

## Data Availability

The data generated in this study are available under study accession PRJEB36273 at the European Nucleotide Archive (www.ebi.ac.uk/ena).

## Supplementary data

**Supplementary Figure S1.** Inference of phylogenetic relationships within *Neurachne*.

**Supplementary Figure S2.** PCA of all *Neurachne* leaf transcripts.

**Supplementary Figure S3.** *Neurachne* species investment in transcripts encoding proteins involved in Cu, S, and N metabolism.

**Supplementary Figure S4.** Cluster analysis of co-expressed genes in *Neurachne* leaf transcriptomes.

**Supplementary Figure S5.** Phylogenetic topologies used for positive selection analyses.

**Supplementary Figure S6.** Amino acid sequence alignment of NADP-ME from *Neurachne* species and selected other NADP-ME grasses.

**Supplementary Table S1.** Transrate results.

**Supplementary Table S2.** RNA-Seq statistics.

**Supplementary Table S3.** Quality assessment of *Neurachne* leaf transcriptome de novo assemblies using the Liliopsida odb10 dataset in BUSCO v.3.0 (Simão et al. 2015; Kriventseva et al. 2019).

**Supplementary Table S4.** Data for the two different topologies resulting from phylogenetic inference of *Neurachne* using ASTRAL and RAxML.

**Supplementary Table S5.** Differentially expressed genes in *Neurachne* leaf transcriptomes.

**Supplementary Table S6.** Alignment information for 12 *Neurachne* C_4_-associated proteins investigated in positive selection analyses.

**Supplementary Table S7.** All results positive selection.

**Supplementary Table S8.** Positively selected sites in two *Neurachne* C_4_-associated proteins.

**Supplementary Table S9.** Predicted disulfide bonds in *Neurachne* C_4_-associated NADP-ME homologs.

**Supplementary Table S10.** *Neurachne* samples used for leaf transcriptome and RT-qPCR analyses.

## Dive Curated Terms

The following phenotypic, genotypic, and functional terms are of significance to the work described in this paper:

MDH Gramene: AT3G47520
MDH Araport: AT3G47520

APS2 Gramene: AT1G05610
APS2 Araport: AT1G05610

APS3 Gramene: AT4G14680
APS3 Araport: AT4G14680

CCH Gramene: AT3G56240
CCH Araport: AT3G56240

TIC21 Gramene: AT2G15290
TIC21 Araport: AT2G15290

CA1 Gramene: AT3G01500
CA1 Araport: AT3G01500

CA2 Gramene: AT5G14740
CA2 Araport: AT5G14740

BUSCO Gramene: Benchmarking Universal Single-Copy Orthologs
BUSCO Araport: Benchmarking Universal Single-Copy Orthologs

UBQ5 Gramene: AT3G62250
UBQ5 Araport: AT3G62250

TPT Gramene: At5g46110
TPT Araport: At5g46110
