# Supplementary material for: Leaf transcriptomes from C3, C3-C4 intermediate, and C4Neurachne species give insights into C4 photosynthesis evolution
Source: Plant Physiol. 2024 Aug 16;197(1):kiae424. doi: 10.1093/plphys/kiae424 (PMC11663609; doi:10.1093/plphys/kiae424)
Supplement: kiae424_Supplementary_Data [file kiae424_supplementary_data.zip › 070824 SUPP FIGS.pdf]

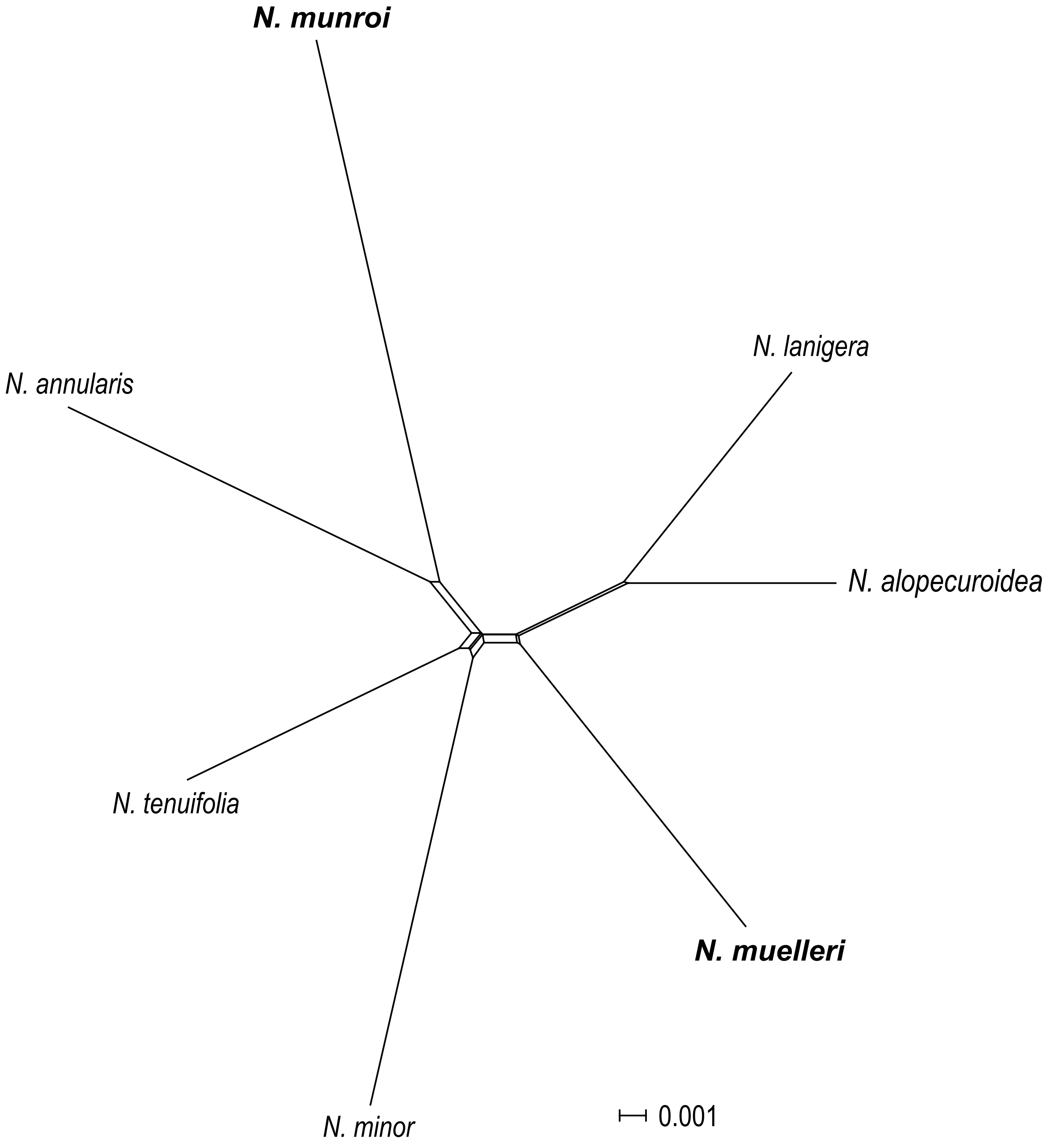

Supplementary Figure S1. Inference of phylogenetic relationships within *Neurachne*. The phylogenetic network was inferred in SplitsTree4 using the Neighbor-Net algorithm (Huson and Bryant, 2006). Names in bold are species that conduct C_4 photosynthesis. Scale bar indicates 0.001 base substitutions per site.

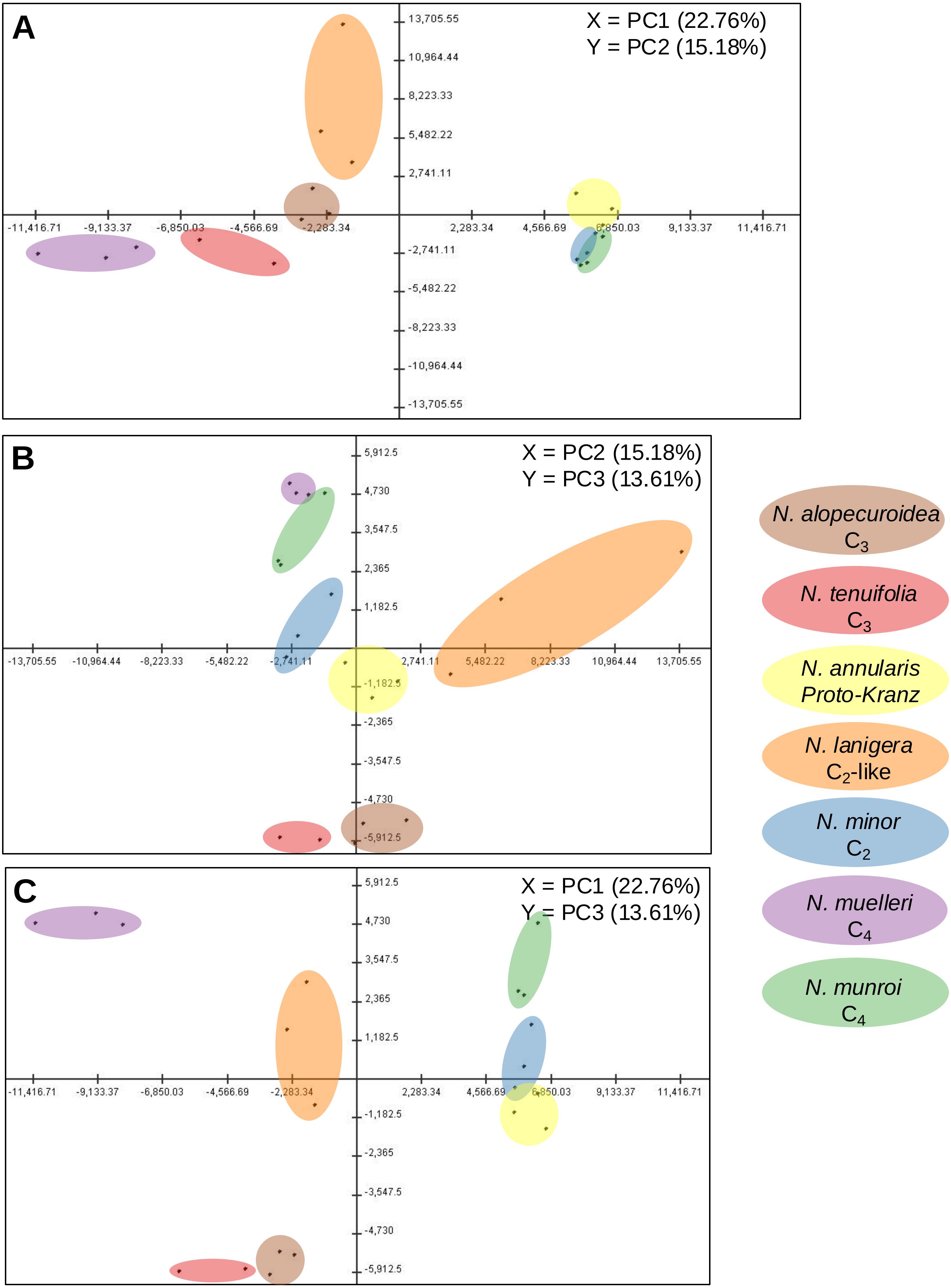

Supplementary Figure S2. Principal component analysis of all *Neurachne* leaf transcripts (transcripts per million values). (A) First (X-axis) and second (Y-axis) components. (B) Second (X-axis) and third (Y-axis) components. (C) First (X-axis) and third (Y-axis) components. First, second, and third components explain 22.76 %, 15.18 %, and 13.61 %, respectively, of the total variation.

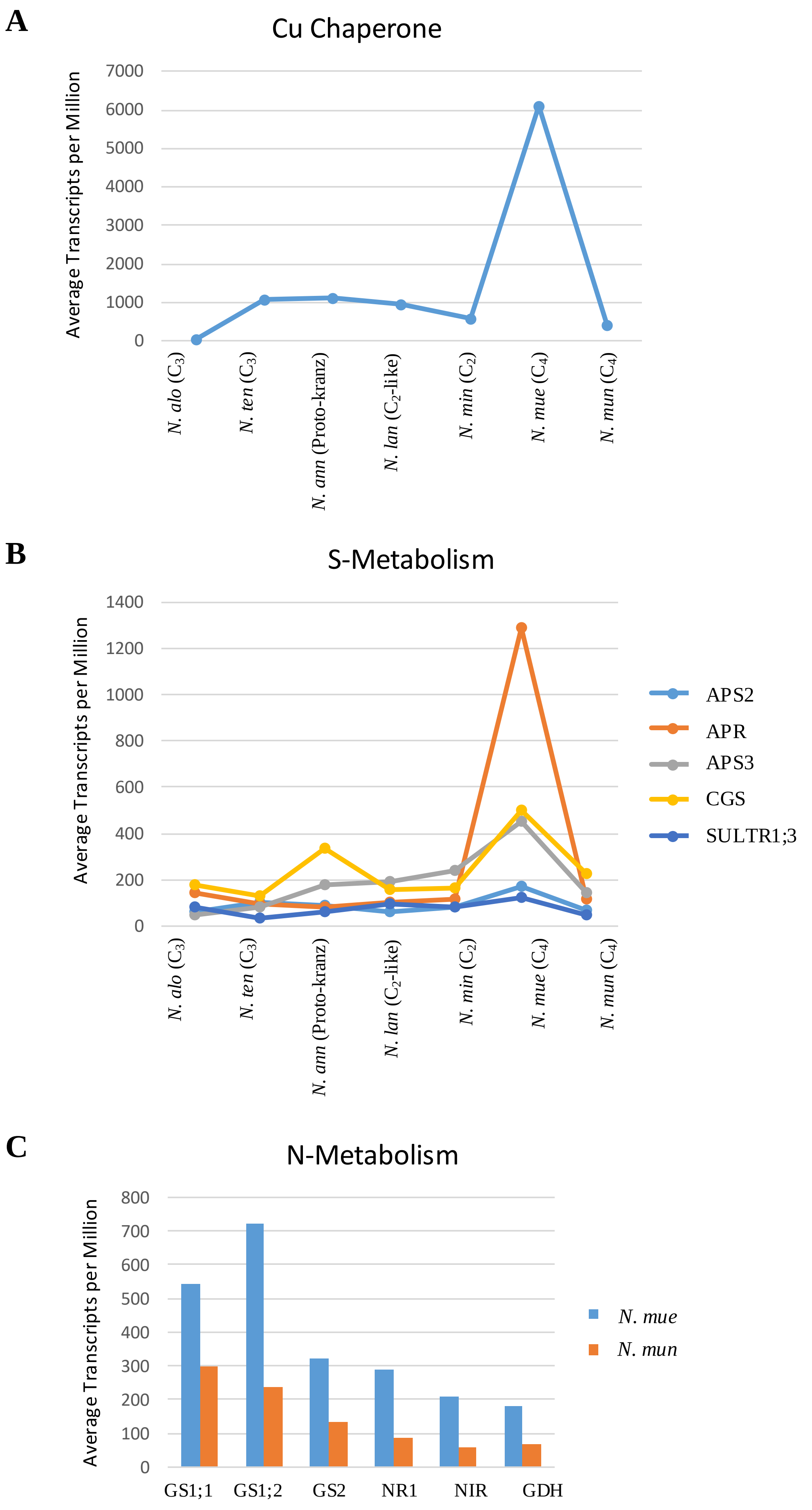
A
Cu Chaperone
Average Transcripts per Million
7000
6000
5000
4000
3000
2000
1000
0
N. alo (C3)
N. ten (C3)
N. ann (Proto-kranz)
N. lan (C2-like)
N. min (C2)
N. mue (C4)
N. mun (C4)
B
S-Metabolism
Average Transcripts per Million
1400
1200
1000
800
600
400
200
0
N. alo (C3)
N. ten (C3)
N. ann (Proto-kranz)
N. lan (C2-like)
N. min (C2)
N. mue (C4)
N. mun (C4)
APS2
APR
APS3
CGS
SULTR1;3
C
N-Metabolism
Average Transcripts per Million
800
700
600
500
400
300
200
100
0
GS1;1
GS1;2
GS2
NR1
NIR
GDH
N. mue
N. mun

Supplementary Figure S3. *Neurachne* species investment in transcripts encoding proteins involved in copper, sulfur, and nitrogen metabolism. Average transcripts per million calculated from three individuals of all *Neurachne* species used in the study, except *N. tenuifolia* (*N. ten*) for which two individuals were available, are shown. (A) Levels of transcripts coding for a copper (Cu) chaperone and (B) proteins involved in sulfur (S) metabolism are highest in C_4 *N. muelleri* (*N. mue*). (C) *N. muelleri* also has a higher abundance of transcripts encoding proteins required for nitrogen (N) metabolism than its C_4 congener *N. munroi* (*N. mun*). *N. alo*, *N. alopecuroidea*; *N. ann*, *N. annularis*; *N. lan*, *N. lanigera*; *N. min*, *N. minor*. APR, adenosine 5'-phosphosulfate reductase; APS2, ATP sulfurylase2; APS3, ATP sulfurylase3; CCH, Cu chaperone; CGS, cystathionine γ-synthase; GDH, glutamine dehydrogenase; GS, glutamine synthetase; NR, nitrate reductase; NIR, nitrite reductase; SULTR1;3, sulfate transporter.

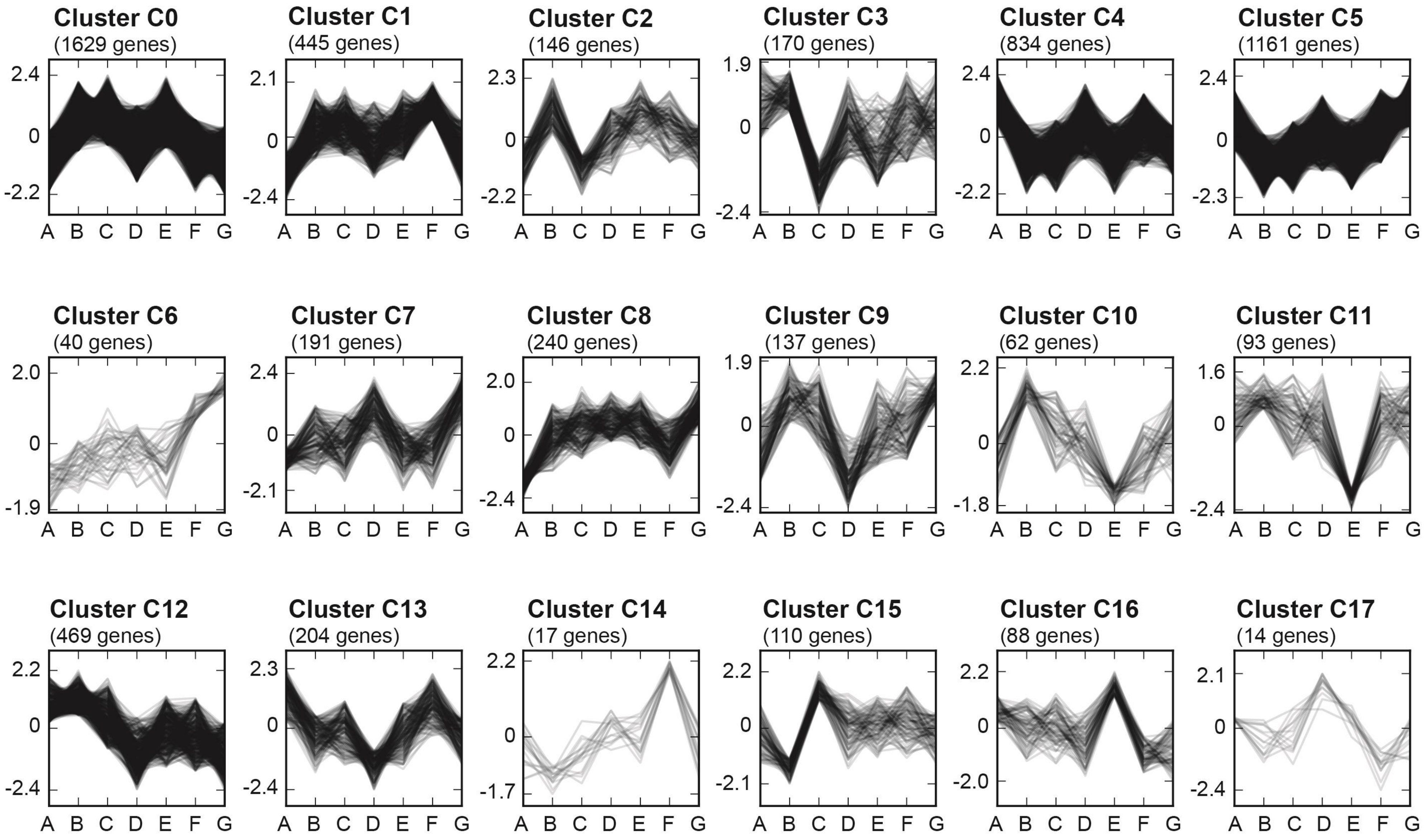

Supplementary Figure S4. Cluster analysis of co-expressed genes in *Neurachne* leaf transcriptomes. Clusters of co-expressed genes were generated using Clust (Abu-Jamous and Kelly, 2018). A total of 6050 genes were grouped into 18 clusters (C1 – C17). A = *Neurachne muelleri* (C_4), B = *N. munroi* (C_4), C = *N. minor* (C_2), D = *N. alopecuroidea* (C_3), E = *N. annularis* (Proto-Kranz), F = *N. lanigera* (C_2-like), and G = *N. tenuifolia* (C_3). *y*-axis, transcript abundance shown as Z-scores.

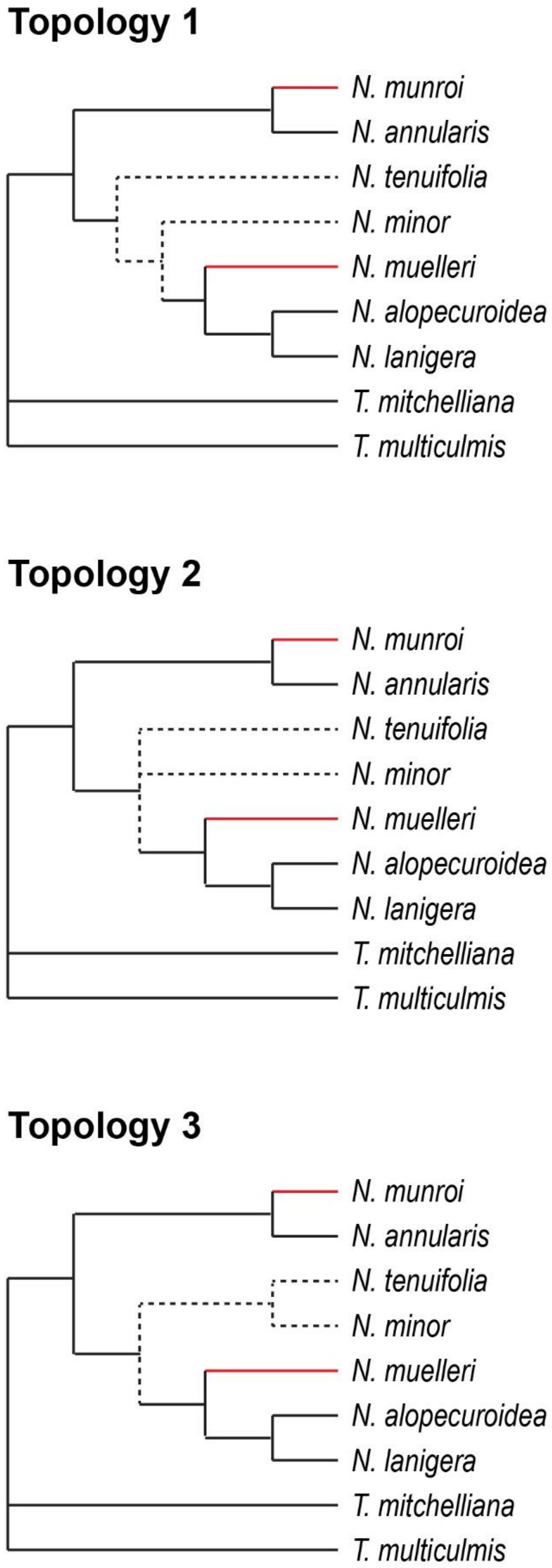

Supplementary Figure S5. Phylogenetic topologies used for positive selection analyses. The three topologies were used in all positive selection analyses since phylogenetic analyses showed incongruences regarding the position of *Neurachne minor* and/or *N. tenuifolia*. C_4 branches (specified as foreground branches) are indicated in red and branches altering between the three topologies are indicated as grey dashes. Trees were forced to have a trifurcation at the root (*Neurachne*, *Thyridolepis mitchelliana*, and *T. multiculmis*), as required by the PAML software.

```
                  10        20        30        40        50        60        70        80        90
N. alo    MLSARTAA---V---------AASPASLWKRGGG-SEGG-SCDGCRTYRESVRRRAAAVRVRAAAPRRVEAVAMGSAAET------EEEK
N. ten    MLSARTA-------------AA-SPAPLWKRGGG-SEGG-SCDGCRTYRESVRRRAAAVRVRAAAPRRVEAVAMGSAAET------EEKE
N. ann    MLSARTA--------------AASPASLWKRGGG-SEGG-SCDGCRTYRESVRRRAAAVLVRAAAPRRVEAVAMGSAAEI------EEEE
N. lan    MLSARTAAA------------AASPASLWKRGGG-SERG-SCDGCRTYRESVRRRAAAVRVRAAAPRRVEAVAMGSAAET------EEEK
N. min    MLSARTA----V--------ASASPASLWKRGGG-S--G-SCDGCRTYRESVRRRAAAVRVRAAARRRAEAVAMGSAVET------EEEE
N. mue    MISSRTAAAATVLSAHTAAAAAASPASLWKRGRG-SPGG-SCDGCRTYRESVRRRAAAARVRAAASRRVEAVAMGTAAETEEKEKEEEKE
N. mun    MLSSNTAAAATVLSARTA-AAAASPASLWKRGGG-SEGG-SCDGCRTYRESARRRAAAARVRAAASRRVDAVAMGTAAETEEKE--EEKE
maize     MLSTRTAA---VAA-------SASPASPWKVGG-RSEGGASCDGCRTYRNTLRRRAAPAKVRALPPRRVDAVAMVSNAET-----ETEKE
sorghum   MISARAAT---VA--------AASPASPWKRGGGRSEGAGSCDGCSTYRKTVRRRAAAAKVRALPPKRVEAVAIGSAAET-----ETEEV
S. ita    MLSARTA----VAS-------AASPASPWKAVGG-SKGG-SCDGCRTYRESVRRRAATVRVHAAEQRRVQAVAVGTAAET----------

                 100       110       120       130       140       150       160       170       180
N. alo    GE-MAAAGGGVEDPYGEDRATEEQPVTPWAFSVASGYTLLRDPHHNKGLAFTEKERDAHYLRGLLPPAVVSQELQIKKIMHNLRQYQVPL
N. ten    EE-MAAAGGGVEDPYGEDRATEEQPVTPWAFSVASGYTLLRDPHHNKGLAFTEKERDAHYLRGLLPPAVVSQELQIKKIMHNLRQYQVPL
N. ann    EE-MAAAGGGVEDPYGEDRATEEQPVTPWAFSVASGYTLLRDPHHNKGLAFTEKERDAHYLRGLLPPAVVSQELQIKKIMHNLRQYQVPL
N. lan    GE-MAAAGGGVEDPYGEDRATEEQPVTPWAFSVASGYTLLRDPHHNKGLAFTEKERDAHYLRGLLPPAVVSQELQIKKIMHNLRQYQVPL
N. min    KEQMAAAGGGIEDPYGEDRATEELPVTPWAFSVASGYTLLRDPHHNKGLAFTEKERDAHYLRGLLPPAVVSQEVQIKKIMHNLRQYQVPL
N. mue    KEDMSAAGGGVDDPYGEDRATEEVPVTPWVYSVASGYALLRDPHYNKALAFTEKERDAHYLRGLLPPAVVPQEIQIKKIMHNLRQYQLPL
N. mun    KEEMAAAGGGVDDAYGEDRATEQVPVTPWVYSVASGYALLRDPHYNKALAFTEKERDAHHLRGLLPPAVVPQEIQIKKIMHNLRQYQLPL
maize     QEEAAAA-------------[illegible]EELPVMPWATYVASGYTLLRDPHHNKGLAFTEEERDGHYLRGLLPPAVLFQELQIKKFMNTLRQYQTPL
sorghum   VVEVAAA-------------NEELPVMPWATSVASGYTLLRDPRHNKGLAFTEKERDAHYLRGLLPPAVVSQELQIKKFMNNLRQYQLPI
S. ita    QEGVAAADGGVVDPYAED---EELPVMPWAYSVASGYTLLRDPHHNKGLAFTEKERDAHYLRGLLPPAVVSQEVQIKKFMHNLRQYQLPL
                                                                                         *
                 190       200       210       220       230       240       250       260       270
N. alo    QRYMAMMDLQERNERLFYKLLIDNVEELLPVVYTPTVGEACQKYGCIFRQPQGLYVSLRDKGKVLEVLRNWPERNIQVIVVTDGERILGL
N. ten    QRYMAMMDLQERNERLFYKLLIDNVEELLPVVYTPTVGEACQKYGCIFRQPQGLYVSLRDKGKVLEVLRNWPERNIQVIVVTDGERILGL
N. ann    QRYMAMMDLQERNERLFYKLLIDNVEELLPVVYTPTVGEACQKYGCIFRQPQGLYVSLRDKGKVLEVLKNWPERNIQVIVVTDGERILGL
N. lan    QRYMAMMDLQERNERLFYKLLIDNVEELLPVVYTPTVGEACQKYGCIFRQPQGLYVSLRDKGKVLEVLRNWPERNIQVIVVTDGERILGL
N. min    QRYMAMMDLQERNERLFYKLLIDNVEELLPVVYTPTVGEACQKYGCIFRQPQGLYVSLRDKGRVLEVLRNWPQRNIQVIVVTDGERILGL
N. mue    QRYMALMDLQERNERLFYKLLIDNVEELLPVVYTPTVGEACQKYGCIFRQPQGLYVSLKDKGRVQEILRNWPQRKIQVICVTDGERILGL
N. mun    QRYMALMDLQERNERLFYKLLIDNVEELLPVVYTPTVGEACQKYGSIFRQPQGLYVSLRDKGRVQEVLRNWPQRKIQVICVTDGERILGL
maize     QRYIAMMNLQETDERLFYKLLIDNVVELLPFVYTPTVGEACQKYGSIFGRPQGLYVSLKDKGKVLEVLRNWPHRNIQVICVTDGERILGL
sorghum   QCYMAMMNLQETDERLFYKLLIENVVELLPYVYTPTVGEACQKYGSIFGRPQGLYVSLKDKGRVLEVLRNWPHRNVQVICVTDGERILGL
S. ita    QRYMAMMDLQERNERLFYRLLIDNVEELLPFVYTPTVGEACQKYGSIFRQPQGLYVSLRDKGRVLEVLRNWPQRDIQVICVTDGGRILGL
                                                                                         *
                 280       290       300       310       320       330       340       350       360
N. alo    GDLGSQGMGIPVGKLALYTALGGVRPSACLPITIDVGTNNEELLNDEFYIGLRQKRATGEEYHELMEEFMNAVKQIYGEKVLIQFEDFAN
N. ten    GDLGSQGMGIPVGKLALYTALGGVRPSACLPITIDVGTNNEELLNDEFYIGLRQKRATGEEYHELMEEFMNAVKQIYGEKVLIQFEDFAN
N. ann    GDLGSQGMGIPVGKLALYTALGGVRPSACLPITIDVGTNNEELLNDEFYIGLRQKRATGEEYHELMEEFMNAVKQIYGEKVLIQFEDFAN
N. lan    GDLGSQGMGIPVGKLALYTALGGVRPSACLPITIDVGTNNEELLNDEFYIGLRQKRATGEEYHELMEEFMNAVKQIYGEKVLIQFEDFAN
N. min    GDLGSQGMGIPVGKLALYTALGGVRPSACLPITIDVGTNNEELLNDEFYIGLRQKRATGEEYHELVEEFMNAVKQIYGEKVLIQFEDFAN
N. mue    GDLGSQGMGIPVGKLALYTALGGVRPSACLPITIDVGTNNEELLNDEFYIGLRQKRATGKEYDELMEEFMNAVKQIYGEKVLIQFEDFAN
N. mun    GDLGSQGMGIPVGKLALYTALGGVRPSACLPITIDVGTNNEKLLNDEFYIGLRQKRATGKEYDELMEEFMSAVKQIYGEKVLIQFEDFAN
maize     GDLGCQGMGIPVGKLALYTALGGVDPSVCLPITIDVGTNNEKLLNDEFYIGLRQKRATGEEYDELIEEFMSAVKQFYGEKVLIQFEDFAN
sorghum   GDLGCQGMGIPVGKLALYTALGGVDPSACLPITIDVGTNNEKLLNDEFYIGLRQKRARGEEYDELMEEFMAAVKTFYGEKVLIQFEDFAN
S. ita    GDLGAQGMGIPVGKLALYTALGGVRPSACLPITIDVGTNNEELLNDEFYIGLRQKRATGKEYHELIEEFMSAVVQIYGEKVLIQFEDFAN
              *
                 370       380       390       400       410       420       430       440       450
N. alo    HNAFDLLAKYSKSHLVFNDDIQGTASVVLAGLLAALKVVGGTLAEHTYLFLGAGEAGTGIAELIALEISKQTKAPIEECRKKVWLVDSKG
N. ten    HNAFDLLAKYSKSHLVFNDDIQGTASVVLAGLLAALKVVGGTLAEHTYLFLGAGEAGTGIAELIALEISKQTKAPIEECRKKVWLVDSKG
N. ann    HNAFDLLAKYSKSHLVFNDDIQGTASVVLAGLLAALKVVGGTLAEHTYLFLGAGEAGTGIAELIALEISKQTKAPIEECRKKVWLVDSKG
N. lan    HNAFDLLAKYSKSHLVFNDDIQGTASVVLAGLLAALKVVGGTLAEHTYLFLGAGEAGTGIAELIALEISKQTKAPIEECRKKVWLVDSKG
N. min    HNAFDLLAKYSKSHLVFNDDIQGTASVVLAGLLAALKVVGGTLAEHTYLFLGAGEAGTGIAELIALEISKQTKAPIEECRKKVWLVDSKG
N. mue    HNAFDLLAKYSKSHLVFNDDIQGTASVVLAGLLAALKVVGGTLAQHTYLFLGAGEAGTGIAELIALEISKQTKAPIEECRKKVWLVDSKG
N. mun    HNAFDLLEKYSKSHLVFNDDIQGTASVVLAGLLAALKMVGGTLAQQTYLFLGAGEAGTGIAELIALEISKQTKAPVEECRKKVWLVDSKG
maize     HNAFDLLEKYSKSHLVFNDDIQGTASVVIAGLLAALKMVGGTLAEQTYLFLGAGEAGTGIAELIALEISKQTNAPLEECRKKVWLVDSKG
sorghum   HNAFDLLEKYSKTHLVFNDDIQGTASVVLAGLLAALKMVGGTLAEQTYLFLGAGEAGTGIAELIALEMSKQTKAPIEECRKKVWLVDSKG
S. ita    HNAFDLLEKYSKSHLVFNDDIQGTASVVLAGLLASLKVVGGTLAEHTYLFLGAGEAGTGIAELIALQISKQTKAPIEECRQKVWLVDSKG
                 *
                 460       470       480       490       500       510       520       530       540
N. alo    LIVNSRKDSLQSFKKPWAHEHEPLTTLFDAVQSIKPTVLIGTSGVGRAFTKEVVEAMASFNERPVIFSLSNPTSHSECTAEEAYNWTQGR
N. ten    LIVNSRKDSLQSFKKPWAHDHEPLTTLFDAVQSIKPTVLIGTSGVGRTFTKEVVEAMASFNERPVIFSLSNPTSHSECTAEEAYNWTQGR
N. ann    LIVNSRKDSLQSFKKPWAHEHEPLTTLFDAVQSIKPTVLIGTSGVGRTFTKEVVEAMASFNERPVIFSLSNPTSHSECTAEEAYNWTQGR
N. lan    LIVNSRKDSLQSFKKPWAHDHEPLTTLLDAVQSIKPTVLIGTSGVGRTFTKEVVEAMASFNERPVIFSLSNPTSHSECTAEEAYNWTQGR
N. min    LIVNSRKDSLQSFKKPWAHDHEPLTTLFDAVQSIKPTVLIGTSGVGRAFTKEVVEAMASFNERPVIFSLSNPTSHSECTAEEAYNWTQGR
N. mue    LIVNSRKDSLQSFKKPWAHEHEPLTTLFDAVQSIKPTVLIGTSGVGRTFTKEVVEAMASFNERPVIFSLSNPTSHSECTAEEAYNWTQGR
N. mun    LLVNSRKDTLQPFKKPWAHEHEPLTTLFDAVQSIKPTVLIGTSGVGRTFTKEVVEAMASFNERPVIFSLSNPTSHSECTAEEAYNWTQGR
maize     LIVDSRKGSLQPFKKPWAHEHEPLKTLYDAVQSIKPTVLIGTSGVGRTFTKEIIEAMSSFNERPIIFSLSNPTSHSECTAEQAYTWSQGR
sorghum   LIVDSRKSSLAPFKKPWAHEHEPLTTLYDAVQSIKPTVLIGTSGVGRTFTKEIVEAMASINERPIIFSLSNPTSHSECTAEQAYTWTQGR
S. ita    LIVSSRKESLPSFKQPWAHEHEPVSTLFDAVQSIKPTVLIGTSGVGRAFTKEVVEAMASFNERPVIFSLSNPTSHSECTAEQAYNWTKGR
                                                                                            *
                 550       560       570       580       590       600       610       620       630
N. alo    AVFASGSPFDPVEYDGKTFVPGQANNAYIFPGFGLGLVISGAIRVHEDMLLAASEALADQATQENFDKGSIFPPFTNIRKISARIAAAVA
N. ten    AVFASGSPFDPVEYDGKTFVPGQANNAYIFPGFGLGLVISGAIRVHEDMLLAASEALADQATQENFDKGSIFPPFTNIRKISARIAAAVA
N. ann    VVFASGSPFDPVEYDGKTFVPGQANNAYIFPGFGLGLVISGAIRVHEDMLLAASEALADQATLENFDKGSIFPPFTNIRKISARIAAAVA
N. lan    AVFASGSPFDLVEYDGKTFVPGQANNAYIFPGFGLGLVISGAIRVHEDMLLAASEALADQATQENFDKGSIFPPFTNIRKISARIAAAVA
N. min    AVFASGSPFDPVEYDGKVFVPGQANNAYIFPGFGLGLVISGAIRVHEDMLLTASEALADQATQENFDKGSIFPPFTNIRKISARIAAAVA
N. mue    AVFASGSPFDPVEYDGKIFVPGQANNAYIFPGFGLGLVISGAIRVHEDMLLAASEALADQATQENFDKGSIFPPFTSIRKISARIAAAVA
N. mun    CVFASGSPFAPVEYNGKTFVPGQANNAYIFPGFGLGLVISGAIRVHEDMLLAASEALADQATQENFDKGSIFPPFTNIRKISAHIAAAVA
maize     SIFASGSPFAPVEYEGKTFVPGQSNNAYIFPGLGLGLVISGAVRVHEDMLLAASKALADQATQDNFEKGSIFPPFTSIRKISAHIAAAVA
sorghum   AVFASGSPFAPVEYDGKTFVPGQSNNAYIFPGLGLGLVISGAVRVHEDMLLAASAALADQATEENFVTGSIFPPFTNIRKISAYIAAAVA
S. ita    AVFASGSPFNPVEYDGKTFVPGQANNAYIFPGLGLGLIMAGATRVHEDMLLAASEALADQANQENFEKGSIFPPFSSIRKISAHIAAAVA
                                          *
                 640       650       660
N. alo    TKAYELGLATRLPPPRDLVKYAESCMYTPVYRNYR
N. ten    AKAYELGLATRLPPPRDLVKYAESCMYTPVYRNYR
N. ann    AKAYELGLATRLPPPRDLVKYAESCMYTPVYRNYR
N. lan    AKAYELGLATRLPPPRDLVKYAESCMYTPVYRIYR
N. min    AKAYELGLATRLPPPRDLVKYAESCMYTPVYRNYR
N. mue    AKAYELGLATRLPPPRDLVKYAESCMYSPIYRNYR
N. mun    AKAYELGLATRLPPPRDLVKYAESCMYTPVYRNYR
maize     AKAYELGLATRLPPPSDLVKYAENCMYTPVYRNYR
sorghum   AKAYELGLATRLPPPKDLVAYAESCMYSPVYRNYQ
S. ita    AKAYELGLATRLPAPRDLVQYAETCMYNPIYRNYR
```

Supplementary Figure S6. Amino acid sequence alignment of NADP-malic enzymes from *Neurachne* species and selected other NADP-ME grasses. Amino acids implicated in maize C_4 NADP-malic enzyme function are indicated by red asterisks (Alvarez and Maurino, 2023) and cysteine residues proposed to be involved in redox regulation are indicated by purple (Alvarez and Maurino, 2023) and orange (current study) asterisks. The region boxed in blue is reported to play a role in oligomerization in maize and sorghum C_4 NADP-ME (Alvarez and Maurino, 2023). *Neurachne alopecuroidea* (*N. alo*, C_3), *N. tenuifolia* (*N. ten*, C_3), *N. annularis* (*N. ann*, Proto-Kranz); *N. lanigera* (*N. lan*, C_2-like); *N. minor* (*N. min*, C_2); *N. munroi* (*N. mun*, C_4); *N. muelleri* (*N. mue*, C_4); *Setaria italica*, *S. ita.* Accession numbers: maize, AY271262.1; sorghum, XM_002454985.2; *S. italica*, XP_004968467.1
