# Supplementary material for: Leaf transcriptomes from C3, C3-C4 intermediate, and C4Neurachne species give insights into C4 photosynthesis evolution
Source: Plant Physiol. 2024 Aug 16;197(1):kiae424. doi: 10.1093/plphys/kiae424 (PMC11663609; doi:10.1093/plphys/kiae424)
Supplement: kiae424_Supplementary_Data [file kiae424_supplementary_data.zip › Supp Table S3S6 S8S10.pdf]

Supplementary Table S3. Quality assessment of *Neurachne* leaf transcriptome *de novo* assemblies using the Liliopsida odb10 dataset in BUSCO v.3.0 (Simão et al., 2015; Kriventseva et al., 2019). BUSCO matches are categorized into complete, fragmented, and missing. Category 'complete' is further subdivided into 'complete and single-copy' and 'complete and duplicated'. The total number of BUSCO genes in the Liliopsida dataset was 3278.

Assembly	Complete		Complete, single		Complete, duplicated		Fragmented		Missing	
	Total	%	Total	%	Total	%	Total	%	Total	%
N. alopecuroidea	2741	83.6	1026	31.3	1715	52.3	246	7.5	291	8.9
N. tenuifolia	2715	82.8	1538	46.9	1177	35.9	196	6.0	367	11.2
N. annularis	2703	82.5	1235	37.7	1468	44.8	283	8.6	292	8.9
N. lanigera	2849	86.9	1575	48.0	1274	38.9	136	4.1	293	9.0
N. minor	2553	77.9	1173	35.8	1380	42.1	364	11.1	361	11.0
N. muelleri	2827	86.2	1508	46.0	1319	40.2	151	4.6	300	9.2
N. munroi	2658	81.1	1159	35.4	1499	45.7	301	9.2	319	9.7

Simão FA, Waterhouse RM, Ioannidis P, Kriventseva E V., Zdobnov EM (2015) BUSCO: Assessing genome assembly and annotation completeness with single-copy orthologs. Bioinformatics **31**: 3210–3212.

Kriventseva E V., Kuznetsov D, Tegenfeldt F, Manni M, Dias R, Simão FA, Zdobnov EM (2019) OrthoDB v10: sampling the diversity of animal, plant, fungal, protist, bacterial and viral genomes for evolutionary and functional annotations of orthologs. Nucleic Acids Res **47**: D807–D811.

Supplementary Table S4. Data for the two different topologies resulting from phylogenetic inference of *Neurachne* using ASTRAL and RAxML. Calculation of gene concordance factors (gCF) and site concordance factors (sCF) for the two different topologies (Fig. 1). Branch support is given in bootstrap (BS) values for tree inference using RAxML and in posterior probability (PP) for tree inference with ASTRAL. gDF_1, gene discordance factor 1; gDF_2, gene discordance factor 2; gN, number of gene trees that could contain the respective branch; sDF_1, site discordance factor 1; sDF_2, site discordance factor 2; sN, average number of sites that could support the respective branch. Nalo, *N. alopecuroidea*; Nann, *N. annularis*; Nlan, *N. lanigera*; Nmin, *N. minor*; Nmue, *N. muelleri*; Nmun, *N. munroi*; Nten, *N. tenuifolia*.

Tree inference method	Branch leading to	gCF		gDF_1		gDF_2		gN	sCF		sDF_1		sDF_2		sN	Branch support (BS/PP)	Branch length
		%	Trees	%	Trees	%	Trees		%	Sites	%	Sites	%	Sites			
ASTRAL	((Nmue,(Nalo,Nlan)),Nmin,Nten)	27.8	558	8.5	170	4.9	99	2004	48.2	6232	25.9	3352	25.9	3348	12931.83	1	0.3425
RAxML	((Nmue,(Nalo,Nlan)),Nmin,Nten)	27.8	558	5.5	110	4.0	81	2004	47.3	6281	27.4	3644	25.3	3363	13288.393	100	0.0029
ASTRAL	(Nalo,Nlan)	71.7	1437	5.9	118	6.5	131	2004	72.3	9048	13.7	1719	14.0	1754	12521.33	1	1.1327
RAxML	(Nalo,Nlan)	71.7	1437	6.5	131	5.9	118	2004	72.2	9060	14.1	1764	13.8	1734	12556.581	100	0.0073
ASTRAL	(Nann,Nmun)	27.3	547	18.1	363	17.4	348	2004	37.3	4758	30.1	3841	32.6	4155	12752.33	1	0.0962
RAxML	(Nann,Nmun)	27.3	547	17.3	348	18.1	363	2004	37.4	4772	32.5	4150	30.1	3847	12769.232	100	0.0019
ASTRAL	(Nmin,(Nmue,(Nalo,Nlan)))	17.5	351	20.1	402	9.8	197	2004	33.3	3628	35.5	3871	31.1	3392	10892.88	0.56	0.0458
RAxML	(Nmin,Nten)	20.1	402	17.5	351	9.8	197	2004	35.9	3854	33.2	3560	30.9	3316	10730.34	79	0.0018
ASTRAL	(Nmue,(Nalo,Nlan))	30.6	614	13.3	268	9.1	182	2004	45.7	5106	28.7	3199	25.6	2860	11164.67	1	0.2604
RAxML	(Nmue,(Nalo,Nlan))	30.6	614	5.8	117	7.7	155	2004	45.5	5188	26.5	3016	28.0	3193	11397.128	100	0.0030

Supplementary Table S5. Differentially expressed genes in *Neurachne* leaf transcriptomes (log_2 fold change ≥1, significance threshold of 0.01 after Benjamini-Hochberg correction).

	N. annularis	*N. lanigera*	*N. minor*	*N. muelleri*	*N. munroi*	*N. tenuifolia*
N. alopecuroidea (C_3)	up: 1264<br>down: 1476	up: 1176<br>down: 1221	up: 1389<br>down: 1598	up: 1537<br>down: 1399	up: 1466<br>down: 1769	up: 948<br>down: 870
N.annularis (Proto-Kranz)		up: 1554<br>down: 1374	up: 944<br>down: 993	up: 2012<br>down: 1860	up: 1012<br>down: 1158	up: 1578<br>down: 1350
N.lanigera (C_2-like)			up: 1438<br>down: 1703	up: 1477<br>down: 1474	up: 1449<br>down: 1841	up: 1300<br>down: 1206
N.minor (C_2)				up: 1966<br>down: 1820	up: 966<br>down: 1078	up: 1546<br>down: 1319
N.muelleri (C_4)					up: 1789<br>down: 2109	up: 1347<br>down: 1383
N.munroi (C_4)						up: 1702<br>down: 1367

Benjamini Y, Hochberg Y (1995) Controlling the false discovery rate: a practical and powerful approach to multiple testing. J R Stat Soc B **57**: 289–300.

Supplementary Table S6. Alignment information for twelve *Neurachne* C_4-associated proteins investigated in positive selection analyses. Alignments included sequence information from seven *Neurachne* species (C_3 *N. alopecuroidea*, C_3 *N. tenuifolia*, proto-Kranz *N. annularis*, C_2-like *N. lanigera*, C_2 *N. minor*, C_4 *N. muelleri*, C_4 *N. munroi*,) and two species of *Thyridolepis* (*T. mitchelliana*, *T. multiculmis*; both C_3). Alignment length is given as nucleotides (bp) and amino acids (aa). The stop codon of each sequence was removed prior to analysis. See Figure 4 for protein abbreviations. Sequence alignments have been submitted to TreeBASE (http://purl.org/phylo/treebase/phylows/study/TB2:S25266).

	**Nucleotide alignment**		**Amino acid alignment**	
**Protein**	**Length (bp)**	**Conserved (%)**	**Length (aa)**	**Conserved (%)**
Ala-AT	1446	91.22	482	93.15
AMK	876	83.11	292	80.14
Asp-AT	1374	88.21	458	93.01
BASS2	1245	88.84	415	87.71
CA1	891	89.45	297	87.54
DiT1	1710	87.37	570	89.82
NADP-MDH	1302	89.63	434	89.17
NADP-ME	1989	88.94	663	87.18
PEPC2	2907	86.65	969	88.96
PEPCK	885	88.47	295	86.10
PPa	867	91.58	289	93.43
PPdK	2841	89.83	947	90.50

Supplementary Table S8. Positively selected sites in two *Neurachne* C_4-associated proteins. Positive selection analyses of phospho*enol*pyruvate carboxylase (PEPC) and NADP-malic enzyme (ME) were based on three different phylogenetic topologies (Supplemental Fig. S5), and Bayes empirical Bayes (BEB; Yang et al., 2005) is given for all three. Amino acid positions with a posterior probability (PP) ≥0.9 of being under positive selection are designated with asterisks: positions with PP ≥0.95, PP ≥0.99, and PP =1 are indicated with one, two and three asterisks, respectively. Sites with the highest posterior probability for each protein are highlighted in bold and were further analyzed. 80+4 refers to the position 4 amino acid residues C-terminal to position 80 of the maize NADP-ME sequence at which gaps were inserted into the maize sequence to maximize the alignment.

	Alignment position	Position maize	BEB			Amino acid C_3 vs C_4
			Topology 1	Topology 2	Topology 3	
PEPC	**242**	**243**	**0.959***	**0.963***	**0.961***	**A/G vs. Y**
	319	320	0.913	0.916	0.915	F vs. I
	530	531	0.924	0.926	0.926	A vs. P
	571	572	0.905	0.908	0.907	E vs. Q
	667	668	0.915	0.918	0.917	L vs. I
	779	780	0.929	0.932	0.931	A vs. S
	793	794	0.916	0.919	0.918	F vs. V/I
	835	836	0.924	0.927	0.926	G vs. A
NADP-ME	4	4	0.969*	0.969*	0.966*	A vs. S
	5	5	0.956*	0.956*	0.952*	R vs. N/R
	36	26	0.957*	0.957*	0.955*	E/- vs. E/P
	57	48	0.955*	0.955*	0.951*	V vs. A
	74	65	0.969*	0.969*	0.966*	S vs. T
	100	(80+4)	0.944	0.944	0.939	E vs. D
	112	85	0.996**	0.996**	0.996**	Q/L vs. V
	118	91	0.957*	0.957*	0.953*	A vs. V
	119	92	0.967*	0.967*	0.964*	F vs. Y
	126	99	0.957*	0.957*	0.953*	T vs. A
	133	106	0.953*	0.953*	0.948	H vs. Y
	136	109	0.969*	0.969*	0.966*	G vs. A
	159	132	0.957*	0.957*	0.953*	S vs. P
	176	149	0.969*	0.969*	0.966*	V vs. L
	184	157	0.963*	0.963*	0.960*	M vs. L
	243	216	0.972*	0.972*	0.970*	L vs. Q
	253	226	0.947	0.947	0.942	N vs. K
	**258**	**231**	**1.000****	**1.000****	**1.000****	**V vs. C**
	331	304	0.961*	0.961*	0.958*	H vs. D
	403	376	0.962*	0.962*	0.959*	E vs. Q

Yang Z, Wong WSW, Nielsen R (2005) Bayes empirical bayes inference of amino acid sites under positive selection. Mol Biol Evol **22**: 1107–1118.

Supplementary Table S9. Predicted disulfide bonds in *Neurachne* C_4-associated NADP-malic enzyme homologs. Disulfide bonds were predicted using DIpro (Baldi et al., 2004; Cheng et al., 2006a, b). Bonds are ordered by probability in descending order. AA, amino acid position; Align, alignment position; NA, not applicable PS, photosynthesis.

Species	PS type	Number of Cys	Predicted number of disulfide bonds	Bond 1		Bond 2		Bond 3	
				Cys 1 (AA /Align)	Cys 2 (AA /Align)	Cys 1 (AA /Align)	Cys 2 (AA /Align)	Cys 1 (AA /Align)	Cys 2 (AA /Align)
Neurachne muelleri	C_4	9	3	40/42	43/45	219/221	224/226	258/260	297/299
N. munroi	C_4	9	3	39/42	42/45	523/528	536/541	255/260	294/299
N. minor	C_2	8	3	26/42	29/45	199/221	204/226	417/439	506/528
N. tenuifolia	C_3	8	3	26/42	29/45	198/221	203/226	416/439	505/528
N. annularis	C_3	8	3	26/42	29/45	198/221	203/226	416/439	505/528
N. lanigera	C_2-like	8	3	28/42	31/45	200/221	205/226	418/439	507/528
N. alopecuroidea	C_3	8	3	28/42	31/45	200/221	205/226	418/439	507/528
Thyridolepis multiculmis	C_3	6	2	162/221	167/226	380/439	469/528	NA	NA
Setaria italica	C_4	8	3	29/42	32/45	195/221	234/260	413/439	502/528
Sorghum bicolor	C_4	10	3	31/42	34/45	246/275	270/299	153/182	192/221
Zea mays	C_4	9	3	31/42	34/45	246/275	270/299	192/221	231/260

Baldi P, Cheng J, Vullo A (2004) Large-scale prediction of disulphide bond connectivity. NIPS'04: Proceedings of the 17th International Conference on Neural Information Processing Systems, pp 97-104.

Cheng J, Randall A, Baldi P (2006a) Prediction of protein stability changes for single-site mutations using support vector machines. Proteins Struct Funct Bioinforma **62**: 1125–1132.

Cheng J, Saigo H, Baldi P (2006b) Large-scale prediction of disulphide bridges using kernel methods, two-dimensional recursive neural networks, and weighted graph matching. Proteins Struct Funct Bioinforma **62**: 617–629.

Supplementary Table S10. *Neurachne* samples used for leaf transcriptome and reverse transcription quantitative polymerase chain reaction analyses. Herbarium abbreviations: DNA, Northern Territory Herbarium; PERTH, Western Australia Herbarium. RT-qPCR, reverse transcription-quantitative polymerase chain reaction analyses; NA, not available.

Species	Number of Individuals		Voucher
	RNA-Seq	RT-qPCR	
Neurachne alopecuroidea	3	1	Sage, TDM5668/ PERTH
	-	2	NA; commercial supplier
Neurachne annularis	1	-	TDM5166 / PERTH
	1	-	TDM5167 / PERTH
	1	-	TDM5381 / PERTH
Neurachne lanigera	3	-	TDM5177 / PERTH
Neurachne minor	2	1	TDM5334 / PERTH
	1	1	TDM5375 / PERTH
	-	1	TDM5175 / PERTH
Neurachne muelleri	3	1	Albrecht 13786 / PERTH
	-	2	TDM6005 / PERTH
Neurachne munroi	1	-	TDM5169 / PERTH
	2	2	TDM5370 / PERTH
	-	1	09582789 / PERTH
Neurachne tenuifolia	2	-	Latz 25064 / DNA
